# Host Plant Biochemical Influence on the Foraging and Food Utilization of *Poecilocerus pictus* and *Danaus chrysippus* on *Calotropis gigantea*: A Behavioral Ecology Approach

**DOI:** 10.3390/insects17090939

**Published:** 2026-09-08

**Authors:** Yewei Jia, Murugan Vasanthakumaran, Rajapandian Rajaganesh, Kadarkarai Murugan, Fajun Chen

**Affiliations:** 1 Department of Entomology, Nanjing Agricultural University (NAU), Nanjing 210095, China; 2025102067@stu.njau.edu.cn; 2 Institute of Marine Biology, National Taiwan Ocean University, Keelung 202301, Taiwan; muruganvasanthakumaran@gmail.com; 3 Department of Zoology, Bharathiar University, Coimbatore 641046, Tamil Nadu, India; tulipsganesh@gmail.com

**Keywords:** *Poecilocerus pictus*, *Danaus chrysippus*, *Calotropis gigantea*, nutrients, cardiac glycosides, amino acids, compensatory feeding

## Abstract

*Poecilocerus pictus* and *Danaus chrysippus* are two common herbivorous insects that share the same host plant, *Calotropis gigantea*, yet they exhibit distinct feeding preferences and food utilization patterns across leaf ages. *D. chrysippus* larvae performed better on young leaves, which contain higher water content and suitable nutrients; before feeding, they cut leaf surfaces and inflorescence bases to interrupt latex flow and access floral sugars. *P. pictus* thrived on mature leaves, which offer a more balanced nutrient composition and moisture level; nymphs made serial cuts on the lower leaf surface to block latex, while adults typically fed from leaf tips. Both species performed poorly on senescent leaves, which are tough, nutrient-poor, and show altered glycoside profiles. Amino acids such as asparagine, proline, glutamine, glutamic acid, valine, alanine, and phenylalanine were more abundant in young leaves, likely explaining the strong preference of *D. chrysippus*, whereas mature leaves had an amino acid profile better suited to *P. pictus*. Gas chromatography–mass spectrometry (GC-MS) analysis revealed distinct phytochemical compositions among young, mature, and senescent leaves, as well as inflorescences. These chemical differences, particularly in water, nitrogen, and key metabolites, strongly influence the feeding behavior and growth of both the hemimetabolous grasshopper and the holometabolous butterfly.

## Simple Summary

*Poecilocerus pictus* and *Danaus chrysippus* are two common insects that feed on wild giant milkweed (*Calotropis gigantea*), a plant known for its toxic latex and strong chemical defenses. Although they share the same host plant, these two species of insects show very different feeding preferences and growth outcomes depending on the age of the leaves they eat. Our study found that *D. chrysippus* grows faster and produces more eggs when feeding on young leaves, which contain more water and nutrients. In contrast, *P. pictus* performs best on mature leaves, which provide a better balance of nutrients and moisture for this grasshopper. Both species of insects struggle to survive on old, tough leaves, which are low in nutrients and high in defensive chemicals. To avoid the plant’s sticky and toxic latex, *P. pictus* and *D. chrysippus* use different strategies that the butterfly larvae of *D. chrysippus* cut leaf veins or flower bases before feeding, while the grasshopper nymphs of *P. pictus* make small cuts along the underside of leaves. Our findings help explain how plant chemistry and physical defenses influence the feeding behavior, growth, and reproduction of herbivorous insects, which is useful knowledge for managing insect pests in agricultural systems.

## 1. Introduction

The nutritional and toxicological ecology of phytophagous insects is fundamentally mediated by the dynamic biochemical and structural profiles of their host plants, which fluctuate substantially across different tissue age classes. The milkweed plant, *Calotropis gigantea*, serves as a high-stakes host for two major herbivorous insects: the painted grasshopper, *Poecilocerus pictus* (Orthoptera: Acrididae), and the African monarch butterfly, *Danaus chrysippus* (Lepidoptera: Nymphalidae). Although these two species share the same host plant, their contrasting life history strategies and developmental programs radically alter how they navigate plant defenses, utilize available nutrients, and allocate resources toward reproduction.

As a hemimetabolous insect, *P. pictus* exhibits continuous, prolonged exposure to the host plant’s defensive chemical arsenal across its entire lifespan. Both nymphs and adults share an identical feeding niche, requiring adaptive physiological buffering and highly specialized cardenolide sequestration mechanisms to handle systemic toxicity over an extended period [1]. Conversely, the holometabolous *D. chrysippus* partitions its dietary niches. The voracious, fast-growing larval stage is dedicated entirely to processing toxic leaf tissues, while the mobile adult stage transitions to nectar-feeding, thereby escaping the structural and chemical defenses of the vegetative tissue [2]. These disparate developmental strategies dictate narrow, high-efficiency growth windows for the butterfly larvae, but allow a more continuous and flexible developmental trajectory for the grasshopper [3].

The underlying driver of these feeding dynamics is the intense chemical and physical heterogeneity found within *C. gigantea* tissues. Recent literature demonstrates that young leaves are generally packed with water and nitrogen—critical structural building blocks for developing insects—but are simultaneously armed with high concentrations of toxic, low-molecular-weight secondary metabolites like cardenolides. In contrast, mature and senescing leaves exhibit reduced nutritional content alongside severe structural physical barriers, such as lignification and leaf toughness, combined with an accumulation of digestibility reducers like phenolics and tannins [4,5].

To survive on this shifting resource landscape, both insects deploy contrasting behavioral and physiological syndromes. Lepidopteran larvae actively prioritize the young, nitrogen-rich tissues to accelerate their growth rates and acquire critical polyunsaturated fatty acids needed for proper pupal development and adult eclosion. When restricted to older, nutritionally diluted foliage, caterpillars display remarkable plasticity by initiating quantitative compensation—eating significantly larger quantities of food and feeding more frequently to meet their basic metabolic targets [6]. They also exhibit elevated digestive enzyme activities and enhanced metabolic detoxification pathway activity, such as cytochrome P450 monooxygenases, to minimize structural tissue inhibition and clear toxic metabolic loads [7].

Orthopterans handle leaf age variance quite differently. Because older leaves feature increased toughness and silica accumulation, chewing grasshoppers face physical barriers that can cause severe mandible wear [8]. Behaviorally, grasshoppers utilize highly sensitive chemosensory sensilla on their maxillary palps to gauge precise carbohydrate-to-protein ratios, allowing them to shift their foraging behavior dynamically [9]. Rather than relying on massive single-tissue intake, they utilize dietary self-selection and mixed-feeding behaviors, combining different leaf ages and inflorescences to complement nutrient deficiencies and dilute localized allelochemical toxins.

To comprehensively map this complex tri-trophic interaction, this study establishes the following six interconnected research objectives: (a) to quantify age-dependent variations in basic nutritional and physical parameters, specifically moisture, nitrogen, total protein, and structural crude fiber, across young, mature, senescent leaves, and inflorescences of *C. gigantean*; (b) to analyze the qualitative and quantitative profiles of essential fatty acids and free amino acid fractions within these distinct host tissue age classes to determine specific nutrient availability; (c) to identify and profile active defensive allelochemicals, including total phenolics and specific cardiac glycoside concentrations, across different tissue stages utilizing advanced GC-MS analysis; (d) to evaluate the direct impacts of variable leaf age on the overall consumption rates, biometric growth dynamics, and larval or nymphal developmental durations of both *P. pictus* and *D. chrysippus*; (e) to determine the physiological influence of host tissue suitability on the midgut digestive enzyme activity and net metabolic conversion efficiencies of both insect species and (f) to assess the long-term, subsequent consequences of larval and nymphal dietary leaf age on adult longevity, reproductive programming, and overall egg fecundity. Through these systematic objectives, this investigation clarifies how host tissue suitability and age-dependent biochemical profiles directly index the reproductive success and compensatory feeding syndromes of specialized hemimetabolous and holometabolous herbivores.

## 2. Materials and Methods

The experimental colonies of the painted grasshopper (*P. pictus*) and the plain tiger butterfly (*D. chrysippus*) were established from field-collected individuals. These founding populations were sourced from wild giant milkweed (*C. gigantea*) populations surrounding the Bharathiar University campus in Coimbatore, Tamil Nadu, India.

### 2.1. Rearing Conditions for Poecilocerus pictus

#### 2.1.1. Environment and Housing

The rearing environment for *P. pictus* was meticulously controlled to ensure standardized conditions for all experimental subjects, with nymphs and newly enclosed adult pairs isolated upon collection and housed in custom-built wooden cages (10 × 20 × 30 cm) (Procured from local hardware supplier, Coimbatore, Tamil Nadu, India) featuring wire-mesh windows on two opposing sides to provide adequate cross-ventilation while securely accommodating the insects’ active behavior. All cages were maintained inside a climate-regulated room where environmental parameters were continuously monitored and stabilized: a 12:12 h light:dark photoperiod was enforced using automated timers coupled with full-spectrum fluorescent lighting (Digital Electronics, Coimbatore, Tamil Nadu, India), temperature was held constant at 28 ± 2 °C, and relative humidity was regulated between 65–70% through ultrasonic humidifiers (Condair Humidifiers, Kolkata, India) adjusted according to real-time digital hygrometer (Thermo Fisher Scientific India Pvt. Ltd., Mumbai, India) readings, thereby minimizing extraneous variability across all rearing stages.

#### 2.1.2. Oviposition Substratum and Reproductive Rearing

To facilitate natural reproductive behavior and ensure the production of stable subsequent generations, each rearing cage was equipped with a plastic oviposition container (10 cm diameter, 8 cm depth) (Thermo Fisher Scientific India Pvt. Ltd., Mumbai, India) firmly embedded at the cage base and filled with a sterilized, moistened sand-soil matrix (1:1 ratio by volume) (Thermo Fisher Scientific India Pvt. Ltd., Mumbai, India), which served as an ideal egg-laying substratum by effectively mimicking the soft soil pockets naturally found beneath wild host plants, thereby encouraging gravid females to oviposit under conditions that closely resembled their native ecological niche.

### 2.2. Rearing Conditions for D. chrysippus

#### 2.2.1. Larval Rearing and Maintenance

To maintain larval cohorts of the specialist lepidopteran *D. chrysippus* under controlled and secure conditions, specimens were reared separately in large, heavy-duty cylindrical plastic containers (width:length:height = 35 cm:40 cm:45 cm), with the mouth of each container tightly secured using fine muslin cloth held in place by high-tension elastic bands—a configuration that ensured adequate, draft-free ventilation while effectively preventing the escape of small, highly mobile neonate larvae throughout their developmental stages.

#### 2.2.2. Dietary Management and Pupation

To ensure optimal nutritional intake and developmental success, larvae were supplied daily with fresh, field-collected *C. gigantea* host leaves ad libitum, with each batch thoroughly washed in distilled water to eliminate any wild detritus or potential predators and subsequently air-dried at room temperature before provisioning; fresh leaf stalks were wrapped in moistened cotton plugs to maintain turgor pressure and leaf quality until complete consumption or replacement at 24-h intervals. This dietary regimen was continued uniformly for all individuals until they reached pupal commitment, which was reliably signaled by a behavioral cessation of feeding and the subsequent formation of characteristic hanging chrysalises along the muslin cloth ceiling, at which point dietary provisioning was no longer required.

#### 2.2.3. Adult Maintenance and Mating Protocols

Following adult emergence, butterfly pairs were gently transferred using fine insect nets to dedicated, spacious mesh flight cages (60 × 60 × 60 cm) designed to provide ample space for natural flight activity and to facilitate successful mating behavior, thereby ensuring optimal conditions for reproductive performance under controlled laboratory settings.

#### 2.2.4. Dietary Support and Sanitation

To sustain adult flight energy and maximize oviposition potential, butterflies were provided with a continuous, clean supply of a 10% sucrose solution delivered via saturated sterile cotton wicks placed in shallow Petri dishes, with these wicks replaced daily as part of a rigorous sanitation routine to eliminate any risk of fungal or microbial contamination, thereby ensuring both nutritional adequacy and hygienic conditions throughout the reproductive period on introduced *C. gigantea* twigs.

### 2.3. Developmental and Fecundity Studies

To systematically evaluate the impact of host tissue quality and developmental aging on life-history traits, neonate first-instar larvae of *D. chrysippus* and freshly hatched first-instar nymphs of *P. pictus* were meticulously isolated from the core colony within 2 h of hatching or emergence and subsequently divided into individual cohorts, each restricted exclusively to one of four distinct tissue classes harvested from wild *C. gigantea* plants: young leaves sourced strictly from apex nodes 1–2 of actively growing branches; mature leaves obtained from fully expanded, dark-green foliage at nodes 5–6; senescent leaves comprising chlorotic, yellowish basal foliage displaying clear signs of natural aging prior to abscission; and intact inflorescences consisting of freshly opened pale-purple umbellate cymes with intact buds and petals, thereby enabling a controlled comparison of dietary effects across varying tissue developmental stages. For each tissue cohort, 30 neonate first-instar larvae of *D. chrysippus* and 30 freshly hatched first-instar nymphs of *P. pictus* were individually isolated (*n* = 30 per treatment per species), yielding a total of 120 individuals per species across the four tissue treatments.

### 2.4. Experimental Setup and Rearing Conditions

To ensure precise monitoring of individual development and to completely eliminate any confounding effects of cannibalism or crowd-induced stress, insects from each tissue cohort were housed singly in transparent, well-ventilated plastic containers (15 × 10 cm), allowing for unambiguous tracking of growth, survival, and developmental milestones under strictly isolated conditions throughout the experimental period.

### 2.5. Data Collection and Monitoring Protocols

Developmental performance was meticulously documented through daily observations conducted at a fixed morning hour (09:00 a.m.), with total body weight accumulation tracked using a microanalytical balance sensitive to 0.1 mg, while the duration of individual post-embryonic instars was precisely determined by collecting cast head capsules and shed exuviae, and total larval or nymphal developmental periods were defined as the cumulative days required from initial hatching to successful pupation (for *D. chrysippus*) or final imaginal molt into an adult (for *P. pictus*). To assess long-term fitness and fecundity under each dietary regime, male-female pairs from identical tissue treatments were paired upon adult emergence, and the following variables were systematically recorded: the pre-oviposition period, measured as the exact time interval in days between adult emergence and the primary egg-laying event; cumulative lifetime egg production, quantified through daily visual inspections of the soil matrix (for grasshoppers) or leaf surfaces (for butterflies) to obtain the total count of eggs deposited per female across her entire lifespan; and total adult longevity, monitored continuously until natural mortality occurred, with data tracked separately for both sexes to detect potential sex-linked dietary compromises. All developmental and fecundity measurements were conducted on 30 individuals per treatment per species (*n* = 30). For adult fecundity assessment, 10 successfully emerged male-female pairs per treatment per species were monitored (*n* = 10 pairs per treatment).

### 2.6. Feeding Preferences and Nutritional Ecology

To comprehensively assess foraging selectivity and the influence of mechanical or chemical tissue traits on orientation, a multiple-choice arena assay was conducted using ten independent trials per insect species, in which five uniform, food-deprived fourth-instar caterpillars of *D. chrysippus* or nymphs of *P. pictus* (starved for 6 h to standardize hunger levels) were simultaneously released into the center of a large, clear glass Petri dish (10 cm diameter) containing alternating, equal-sized leaf disks (2 cm diameter) precisely cut from young, mature, and senescent leaves using a sharp brass cork borer while carefully avoiding thick primary midribs to ensure structural uniformity, with disks pinned equidistantly along the perimeter; insect positioning and initial feeding strikes—defined as sustained mandibular contact and tissue removal exceeding 1 min—were recorded during continuous observation, and total tissue displacement was documented over a 3-to-6-h window to eliminate individual behavioral bias. For the multiple-choice arena assay, ten independent trials were conducted per insect species (*n* = 10 dishes per species), with each dish containing five insects. For quantitative nutritional efficiency evaluation, nine newly molted fourth-instar insects per leaf treatment per species were individually weighed and monitored (*n* = 9 per treatment per species). Preference data were analyzed using one-way ANOVA with tissue type as the fixed factor, followed by DMRT for mean separation (*p* < 0.05). This dish-level analysis avoids pseudo-replication by using independent experimental units rather than individual insect responses within a dish. Quantitative nutritional efficiency was subsequently evaluated gravimetrically following Waldbauer [10] and Slansky and Scriber [11], whereby solitary, newly molted fourth-instar insects were weighed and transferred into individual sealed feeding chambers containing pre-weighed leaf treatment aliquots, with control leaves (without insects) maintained under identical conditions to calculate natural moisture loss, and all samples were oven-dried to establish wet and dry mass values for computing the standard indices:

Consumption Index (CI) = *F*/(*A* × *T*)

Relative Growth Rate (RGR) = *G*/(*A* × *T*)

Approximate Digestibility (AD) = ((*F* − *E*)/*F*) × 100

Efficiency of Conversion of Ingested Food (ECI) = (*G*/*F*) × 100

Efficiency of Conversion of Digested Food (ECD) = (*G*/(*F* − *E*)) × 100

where *F* = dry weight of food consumed; *G* = dry weight gain of the insect; *E* = dry weight of feces excreted; *A* = mean dry weight of the insect during the feeding period calculated as (initial dry weight + final dry weight)/2; *T* = duration of the experimental feeding period (1 day).

To further determine internal processing and egestion kinetics under varying fiber and cardenolide loads, separate cohorts of nine insects per leaf treatment were monitored continuously, with the exact time from initial feeding to evacuation of the first colored fecal pellet recorded as gut transit time, alongside the total volume and count of pellets produced over a full 24-h cycle, thereby providing a comprehensive profile of both behavioral preference and physiological processing efficiency across tissue types.

### 2.7. Digestive Enzyme Extraction and Assays

#### 2.7.1. Tissue Harvesting and Dissection

To investigate digestive physiology, midgut tissue matrices were harvested from actively feeding fifth-instar nymphs of *P. pictus* and caterpillars of *D. chrysippus* during their peak linear growth phase, with all dissections performed under a stereo binocular microscope in ice-cold insect Ringer’s solution (NaCl 130 mM, KCl 5 mM, CaCl_2_ 1 mM) (Sigma-Aldrich, St. Louis, MO, USA ≥95% purity) to preserve enzyme stability and prevent proteolysis (*n* = 5 per treatment per species). The midgut walls were carefully slit longitudinally, and the peritrophic membranes along with any trapped food boluses were discarded, after which the isolated tissues were rinsed in fresh buffer, blotted on filter paper, and homogenized in cold citrate-phosphate buffer (Sigma-Aldrich, St. Louis, MO, USA) optimized to pH 6.8 using a motorized Teflon-glass pestle. The raw homogenates were then centrifuged at 10,000× *g* for 20 min at 4 °C in a refrigerated centrifuge, and the clear, lipid-free intermediate supernatant was collected with a micropipette (Sigma-Aldrich, St. Louis, MO, USA) to serve as the crude enzyme source for subsequent biochemical assays. All specific enzyme activities were normalized and expressed as units ×10^−3^ per milligram of dry tissue weight per minute. One unit of amylase activity was defined as the amount of enzyme liberating 1 μmol of glucose equivalents per minute at 37 °C; one unit of protease activity was defined as the amount of enzyme releasing 1 μmol of tyrosine equivalents per minute at 37 °C; and one unit of lipase activity was defined as the amount of enzyme liberating 1 μmol of free fatty acid equivalents per minute at 37 °C. The total reaction volumes were 1.6 mL (amylase), 2.2 mL (protease), and 5.5 mL (lipase).

#### 2.7.2. Amylase Activity Assay

Amylase activity was determined spectrophotometrically using the dinitrosalicylic acid (DNS) method. The assay mixture comprised 0.5 mL of 2% (*w*/*v*) soluble starch substrate and 0.1 mL of crude enzyme supernatant, incubated at 37 °C for 30 min. The reaction was terminated by adding 1.0 mL of alkaline DNS reagent, followed by heating in a boiling water bath for exactly 5 min to develop the chromophore. After cooling, the mixture was diluted with 5 mL of distilled water, and absorbance was measured at 550 nm against an enzyme-free blank. Enzyme activity was quantified using a standard curve constructed with purified D-glucose. The total reaction volume was 1.6 mL (0.5 mL of 2% soluble starch substrate +0.1 mL crude enzyme supernatant +1.0 mL DNS reagent added after incubation). This volume was consistent across all replicates. (One unit of amylase activity is defined as the amount of enzyme that liberates 1 μmol of maltose (or glucose equivalent) per minute under the assay conditions (37 °C, pH 6.8, 30-min incubation). Activity was calculated using a D-glucose standard curve and expressed as units ×10^−3^ per mg of dry tissue weight per minute.)

#### 2.7.3. Protease Activity Assay

Proteolytic activity was assayed using the Folin-phenol copper reduction method, optimized for alkaline digestive conditions. The reaction mixture contained 0.2 mL of crude enzyme extract and 1% (*w*/*v*) bovine serum albumin (BSA) dissolved in glycine-NaOH buffer (pH 11.7) incubated at 37 °C for 60 min. The enzymatic reaction was terminated by adding 2.0 mL of 10% (*w*/*v*) trichloroacetic acid (TCA), and precipitated proteins were removed by centrifugation at 5000× *g* for 10 min. The resulting supernatant was treated with Folin–Ciocalteu phenol reagent and copper solution, and absorbance was recorded at 600 nm against tyrosine equivalents as the reference standard. The total reaction volume was 2.2 mL (0.2 mL crude enzyme extract +1% BSA in glycine-NaOH buffer, volume adjusted to 2.0 mL total, with 2.0 mL TCA added to terminate the reaction). (One unit of protease activity is defined as the amount of enzyme that releases 1 μmol of tyrosine equivalents per minute under the assay conditions (37 °C, pH 11.7, 60-min incubation). Activity was quantified using a tyrosine standard curve and expressed as units ×10^−3^ per mg of dry tissue weight per minute.).

#### 2.7.4. Lipase Activity Assay

Lipase activity was measured via micro-titration, based on the hydrolysis of a stabilized olive oil emulsion. The assay mixture, buffered to pH 8.0 with Tris-HCl, was incubated at 37 °C for 24 h. Liberated free fatty acids were quantified by manual micro-titration against 0.05 N sodium hydroxide (NaOH) using 1% phenolphthalein as the indicator, with the endpoint determined by the appearance of a persistent pink coloration. All specific enzyme activities were normalized and expressed as units × 10^−4^ per milligram of dry tissue weight per minute. The assay mixture consisted of 5 mL of olive oil emulsion in Tris-HCl buffer (pH 8.0) with 0.5 mL crude enzyme extract. (One unit of lipase activity is defined as the amount of enzyme that liberates 1 μmol of free fatty acid (as oleic acid equivalent) per minute under the assay conditions (37 °C, pH 8.0, 24-h incubation). Activity was calculated from NaOH titration and expressed as units × 10^−3^ per mg of dry tissue weight per minute.).

### 2.8. Phytochemical Screening and Biochemical Quantification

#### 2.8.1. Macro-Nutritional and Protein Components

##### Total Carbohydrates

Soluble sugar fractions were extracted from oven-dried plant tissue powder and quantified calorimetrically using the phenol-sulfuric acid method. Briefly, 1 mL of plant tissue extract was sequentially treated with 1 mL of 80% aqueous phenol and 5 mL of concentrated H_2_SO_4_. The exothermic reaction facilitated complete dehydration, yielding a stable golden-yellow furfural complex. After cooling for 30 min, absorbance was measured at 490 nm against a D-glucose standard curve.

##### Total Nitrogen Content

Total nitrogen was determined using the automated micro-Kjeldahl digestion and distillation technique. Finely ground dry tissue (0.2 g) was digested in heavy-walled glass tubes with 5 mL of concentrated H_2_SO_4_ and a K_2_SO_4_–CuSO_4_ catalyst mixture until a clear green digest was obtained. The digest was subjected to alkaline steam distillation with 40% NaOH into a receiver containing 10 mL of 2% boric acid and a mixed methyl red–methylene blue indicator. The trapped ammonia was quantified by back-titration against 0.1 N HCl.

##### Total Soluble Proteins

Protein content was estimated following the Folin-phenol copper reduction assay. Tissue extracts were reacted with alkaline cupric tartrate reagent to form copper–peptide coordination complexes, which subsequently reduced the phosphomolybdic–phosphotungstic acid component of Folin–Ciocalteu reagent, producing a deep blue chromophore. Absorbance was measured at 750 nm and calibrated against a bovine serum albumin (BSA) standard curve.

##### Total Amino Acids

Total free amino acids were determined spectrophotometrically using the ninhydrin colorimetric method in a 0.2 M sodium citrate buffer system (pH 5.0). The reaction mixtures were heated in a boiling water bath for 35 min to develop the characteristic purple Ruhemann’s complex, rapidly cooled, and absorbance read at 570 nm, with DL-alanine serving as the primary calibration standard.

#### 2.8.2. Mineral and Structural Properties

##### Phosphorus and Potassium Content

Ground vegetative samples were dried to constant mass at 70 °C and incinerated in a muffle furnace at 500 °C for 6 h to eliminate organic matter. The resultant mineral ash was dissolved in 5 mL of 0.1 N HCl. Potassium was quantified using atomic absorption spectrophotometry, while phosphorus was determined colorimetrically at 882 nm via the ammonium molybdate–antimony potassium tartrate–ascorbic acid reduction method.

##### Moisture and Crude Fiber

Moisture content was calculated gravimetrically from the mass difference of 2 g fresh tissue before and after drying at 37 °C until constant weight. Crude fiber was estimated following standard acid-detergent and alkali-digestion extraction protocols to isolate insoluble lignocellulosic fractions.

#### 2.8.3. Secondary Metabolites and Allelochemicals

##### Total Phenolics

Soluble phenolics were extracted in acidified methanol and reacted with Folin–Ciocalteu reagent under alkaline conditions (20% Na_2_CO_3_). The resulting blue molybdenum reduction complex was measured at 725 nm and quantified against a catechol standard curve.

##### Cardiac Glycosides (Cardenolides)

Total cardenolide concentrations were determined based on the interaction of the characteristic α,β-unsaturated lactone ring with alkaline 3,5-dinitrobenzoic acid. To eliminate spectral interference, chlorophyll and carotenoid pigments were removed from methanolic extracts using C-18 solid-phase extraction (SEP-PAK) cartridges prior to color development. To eliminate spectral interference from chlorophyll and carotenoid pigments, methanolic extracts were passed through C-18 solid-phase extraction (SEP-PAK) cartridges (Waters, Milford, MA, USA, 500 mg, 6 mL). Cartridges were preconditioned with 5 mL of methanol followed by 5 mL of deionized water. The sample (2 mL in 50% aqueous methanol) was loaded, and pigments were eluted with 5 mL of hexane followed by 5 mL of 20% ethyl acetate in hexane. Cardiac glycosides were then eluted with 8 mL of methanol. The methanol eluate was collected, evaporated to dryness under a stream of nitrogen, and reconstituted in 1 mL of methanol for colorimetric analysis. Absorbance was recorded at 565 nm and converted to micrograms of ouabain equivalents per 0.1 g dry tissue weight. Cardiac glycosides (cardenolides) were quantified based on the interaction of the characteristic α,β-unsaturated lactone ring with alkaline 3,5-dinitrobenzoic acid (DNBA). Under alkaline conditions, the lactone ring undergoes nucleophilic addition, forming a colored complex with maximum absorbance at 565 nm. The assay was calibrated using ouabain (Sigma-Aldrich, St. Louis, MO, USA ≥95% purity) as the reference standard, with concentrations expressed as μg ouabain equivalents per 100 mg dry tissue weight. A standard curve was constructed using ouabain concentrations ranging from 0 to 200 μg/mL.

##### Free Amino Acid Profiling

Individual free amino acids were separated and identified using automated high-resolution ion-exchange chromatography on a Yanaco LC-58 Amino Acid Analyzer, (Yanaco India Private Limited, Bengaluru, India) employing a lithium/sodium citrate step-elution buffer system. Tryptophan was selectively resolved on a Sephadex G-10 molecular sieve column and confirmed fluorometrically following post-column derivatization with Ehrlich’s reagent.

#### 2.8.4. GCMS Analysis

Methanolic extracts of young leaves, mature leaves, senescent leaves, and inflorescences of *C. gigantea* were subjected to gas chromatography–mass spectrometry (GC-MS) analysis for identification and quantification of bioactive compounds. Briefly, dried powdered plant material (5 g) was extracted with 50 mL of methanol (HPLC grade) through continuous agitation for 24 h at room temperature, followed by filtration and solvent evaporation under reduced pressure. The resulting residues were reconstituted in 1 mL of methanol and filtered through a 0.22 μm syringe filter prior to injection. GC-MS analysis was performed on a system equipped with a capillary column (e.g., DB-5MS, 30 m × 0.25 mm × 0.25 μm) coupled to a mass selective detector. Helium served as the carrier gas at a flow rate of 1 mL/min, with an injection volume of 1 μL in splitless mode. The oven temperature program was initiated at 70 °C, ramped to 280 °C at 10 °C/min, and held for 10 min. Mass spectra were recorded at 70 eV over a mass range of *m*/*z* 40–650. Compound identification was achieved by comparing retention indices and mass fragmentation patterns with the NIST/Wiley spectral libraries.

### 2.9. Statistical Analysis

All quantitative data were expressed as mean ± standard error of the mean (S.E.M.). Differences among tissue classes and insect performance metrics were evaluated using one-way analysis of variance (ANOVA), with significant mean separations determined by Duncan’s Multiple Range Test (DMRT) at a significance threshold of *p* < 0.05. For adult longevity and reproductive parameters, sex was included as an additional fixed factor in the ANOVA model. The interaction between tissue type and sex was not significant for any adult trait (*p* > 0.05 for all comparisons). Sex-specific adult longevity data are provided in Supplementary Table S1.

## 3. Results

### 3.1. Biological and Reproductive Parameters of P. pictus and D. chrysippus Fed on Different Host Plant Parts

The biological and reproductive parameters of *P. pictus* and *D. chrysippus* vary significantly depending on the age of the leaves and the inflorescence of *C. gigantea* supplied during development (Table 1 and Table 2).

#### 3.1.1. Biological and Reproductive Parameters of *P. pictus*

Dietary regimes significantly alter the nymphal development and adult reproductive performance of *P. pictus*. As detailed in Table 1, the total nymphal duration across the five instars shows clear developmental acceleration when insects are reared on mature leaves, requiring only 49.00 days. Adult longevity trends show that mature leaves extend lifespans to 116.23 days for males and 140.70 days for females. Conversely, senescent leaves reduce adult survival to 88.76 days for males and 109.84 days for females. The sex-specific longevity patterns were consistent across all tissue treatments, with females consistently outliving males regardless of diet (Supplementary Table S1). One-way ANOVA with sex as an additional fixed factor confirmed that the interaction between tissue type and sex was not significant for longevity in either species (*P. pictus*: F_3,72_ = 1.45, *p* = 0.235; *D. chrysippus*: F_3,72_ = 1.05, *p* = 0.376), indicating that sex did not confound the observed treatment effects (Supplementary Table S1).

Development is delayed on young leaves (55.51 days) and inflorescences (56.57 days), and it becomes severely extended on senescent leaves, taking 63.98 days. This developmental trend is consistent across individual instars: I Instar: Ranges from a swift 10.00 days on mature leaves to 12.40 days on senescent leaves. V Instar: Requires 10.29 days on mature leaves but extends to 14.80 days on senescent leaves. Adult reproductive markers and longevity display a similar pattern based on host plant tissue suitability. The pre-oviposition period is shortest for females fed mature leaves (20.50 days) and longest for those restricted to senescent leaves (29.36 days). The actual windows for egg-laying and post-egg-laying expand on an optimal diet; mature leaves yield an oviposition period of 52.20 days and a post-oviposition period of 29.56 days. On senescent leaves, these periods drop significantly to 15.25 days and 11.28 days, respectively. Adult longevity trends show that mature leaves extend lifespans to 116.23 days for males and 140.70 days for females. Conversely, senescent leaves reduce adult survival to 88.76 days for males and 109.84 days for females. Total fecundity is directly affected by these dietary inputs. Peak egg production occurs on mature leaves, yielding an average of 511.96 eggs per female. This drops significantly to 434.61 eggs on inflorescences, 407.17 eggs on young leaves, and a low of 271.75 eggs on senescent leaves. Consequently, the suitability order for *P. pictus* is: mature leaves > young leaves > inflorescence > senescent leaves.

#### 3.1.2. Biological and Reproductive Parameters of *D. chrysippus*

The dietary requirements of the holometabolous butterfly *D. chrysippus* contrast with those of *P. pictus*, as shown by the life history metrics in Table 2. Larval development is fastest on young leaves, with a total larval duration of 10.12 days. Developmental timelines lengthen progressively on inflorescences (11.36 days), mature leaves (12.06 days), and senescent leaves (14.38 days). Adult longevity also depends heavily on larval nutrition. Young leaves extend adult lifespans to 9.56 days for males and 10.48 days for females. Mature leaves drop these values to 5.34 days for males and 4.78 days for females, while senescent leaves reduce adult survival to 3.38 days and 3.34 days, respectively. The sex-specific longevity patterns are presented in Supplementary Table S1, confirming that the observed treatment effects on adult survival were consistent across sexes (Sex × Tissue interaction: F_3,72_ = 1.05, *p* = 0.376). The suitability order for *D. chrysippus* is: young leaves > inflorescence > mature leaves > senescent leaves.

The prepupal period is shortest on young leaves at 2.26 days, extending to 3.22 days on senescent foliage. The pupal period is completed in 9.50 days on young leaves, while it is 11.54 days on senescent leaves. Upon emergence, the pre-oviposition period for adult females is shortest in the young leaf group (3.11 h) and longest in the senescent leaf group (4.16 h). The oviposition period is extended to 23.07 h on young leaves but drops to 13.86 h on mature leaves and 6.18 h on senescent leaves. Fecundity drops significantly when larvae are fed sub-optimal tissues; females from the young leaf group lay a maximum of 162.70 eggs, whereas those from the senescent leaf group average just 60.16 eggs. Adult longevity also depends heavily on larval nutrition. Young leaves extend adult lifespans to 9.56 days for males and 10.48 days for females. Mature leaves drop these values to 5.34 days for males and 4.78 days for females, while senescent leaves reduce adult survival to 3.38 days and 3.34 days, respectively. The suitability order for *D. chrysippus* is: young leaves > inflorescence > mature leaves > senescent leaves.

### 3.2. Quantitative Food Intake and Feeding Behavior of P. pictus and D. chrysippus Fed on Different Host Plant Parts

Quantitative food consumption varies across developmental stages and host plant tissue types for both species (Table 3 and Table 4). Food intake increases as life stages advance. In *P. pictus*, consumption rises continuously from the first instar up to the initial oviposition period, with the highest food intake occurring during egg-laying. Individuals fed mature leaves exhibit higher quantitative consumption and biomass weight gain than those on other diets. For *D. chrysippus*, food consumption peaks during the third and fourth instars, with the absolute maximum intake occurring in the fourth instar. Total food intake is consistently higher on young leaves than on mature or senescent foliage.

#### 3.2.1. Feeding Behaviors Match These Preferences over Time

*P. pictus* Nymphs: First instars feed exclusively on inflorescences and young leaves. Second and third instars prefer young and mature leaves, while later instars feed on a mix of tissues. They consume leaves from the margins (tip to bottom) and make small cuts along the backside of the leaf midrib to stop latex flow.

*D. chrysippus* Larvae: First instars feed exclusively on inflorescences. Second and third instars focus on young leaves, chewing irregular pits between the side ribs. Fourth and fifth instars consume fully mature leaves and inflorescences along the leaf margins. They cut the main leaf rib before chewing to disrupt latex flow. When feeding on flowers, they selectively eat the calyx and corolla while avoiding the reproductive organs.

The latex-circumventing tactics of the two species differ in several respects. Nymphs of *P. pictus* cut the lower surface of the midrib repeatedly, whereas larvae of *D. chrysippus* ever the main leaf vein with a single cut before feeding. The grasshopper feeds from the leaf tip toward the base; the butterfly feeds from the margin or from the cut site depending on leaf age. Moreover, *P. pictus* nymphs do not feed on inflorescences, while *D. chrysippuslarvae* consume the calyx and corolla but avoid the reproductive organs. Vein-cutting is performed by *P. pictus* throughout all nymphal instars, but in *D. chrysippus* it is most evident during the fourth and fifth instars. These contrasting behaviors reflect the distinct evolutionary pathways by which a hemimetabolous grasshopper and a holometabolous butterfly have adapted to the pressurized latex system of *C. gigantea*.

#### 3.2.2. Food Utilization Efficiencies of Fifth Instar *P. pictus*

Food utilization efficiencies in fifth-instar nymphs of *P. pictus* varied considerably across host tissues of *C. gigantea* (Table 3). The Consumption Index (CI) ranged from 0.271 g (mature leaves) to 0.315 g (young leaves), with young leaves (0.315 g) and inflorescences (0.310 g) recording significantly higher values compared to mature leaves (0.271 g), while senescent leaves (0.302 g) exhibited intermediate values that did not differ significantly from inflorescences. Relative Growth Rate (RGR) followed a similar trend, being highest on young leaves (0.077 g) and inflorescences (0.076 g), followed by senescent leaves (0.071 g), and lowest on mature leaves (0.064 g). Approximate Digestibility (AD) was markedly superior on mature leaves (55.96%), which was substantially higher than that recorded on inflorescences (47.16%), young leaves (45.15%), and senescent leaves (48.91%), indicating that fully expanded foliage was assimilated more efficiently. In contrast, the Efficiency of Conversion of Ingested Food (ECI) remained statistically comparable across all dietary treatments, with values fluctuating narrowly between 23.83% (senescent leaves) and 24.83% (inflorescences), suggesting that ingested material was converted to biomass with similar overall efficiency irrespective of tissue type. The Efficiency of Conversion of Digested Food (ECD), however, revealed a converse pattern, with the highest values observed on young leaves (54.32%) and inflorescences (52.66%), followed by senescent leaves (48.74%), and the lowest on mature leaves (42.62%), implying that while mature leaves were highly digestible, the conversion of digested nutrients into body mass was least efficient on this tissue. Collectively, these results demonstrate that mature leaves optimize assimilation but compromise post-digestive conversion, whereas young leaves and inflorescences provide a more favorable balance between ingestion, digestion, and conversion, whereas senescent leaves sustain moderate but suboptimal performance across most indices.

#### 3.2.3. Food Utilization Efficiencies of Fifth Instar *D. chrysippus*

Food utilization efficiencies in fifth-instar larvae of *D. chrysippus* exhibited pronounced variation across different host tissues of *C. gigantea* (Table 4). The Consumption Index (CI) was significantly highest on young leaves (0.458 g) and inflorescences (0.416 g), followed by mature leaves (0.351 g), and lowest on senescent leaves (0.280 g), indicating a marked feeding preference for younger, metabolically active tissues. Relative Growth Rate (RGR) followed a similar gradient, with maximal values recorded on young leaves (0.110 g), substantially exceeding those on inflorescences (0.073 g), mature leaves (0.053 g), and senescent leaves (0.041 g), reflecting superior biomass accretion on the most nutritionally favorable diets. Approximate Digestibility (AD) displayed a contrasting pattern, being highest on senescent leaves (54.19%), followed by young leaves (48.15%), inflorescences (46.98%), and mature leaves (45.31%), suggesting that despite lower overall nutritional quality, senescent tissues were assimilated with greater efficiency, possibly due to reduced soluble inhibitory compounds or altered cell wall architecture. The Efficiency of Conversion of Ingested Food (ECI) was significantly elevated on young leaves (24.18%) compared to inflorescences (17.67%), mature leaves (15.35%), and senescent leaves (14.89%), indicating that ingested material from young foliage was converted into larval biomass most effectively. Similarly, the Efficiency of Conversion of Digested Food (ECD) was highest on young leaves (50.22%), followed by inflorescences (37.62%), mature leaves (33.88%), and lowest on senescent leaves (27.48%), revealing that post-assimilatory conversion efficiency was markedly superior on young tissues. Collectively, these results demonstrate that young leaves represent the optimal dietary resource for *D. chrysippus*, supporting the highest ingestion, growth, and conversion efficiencies, while inflorescences serve as a competent alternative, and mature and senescent leaves progressively diminish nutritional performance across all measured indices.

### 3.3. Digestive Enzyme Profile of P. pictus and D. chrysippus

#### 3.3.1. Digestive Enzyme Profile of *P. pictus*

Quantitative analysis of digestive enzyme activities in fifth-instar nymphs of *P. pictus* fed on different host parts of *C. gigantea* revealed significant variations (*p* < 0.05) across dietary treatments (Table 5). Maximum enzymatic activities for all three classes—proteases (102.05 ± 1.57 × 10^−3^ units), amylases (99.98 ± 1.14 × 10^−3^ units), and lipases (4.02 ± 1.00 × 10^−3^ units)—were recorded in nymphs consuming mature leaves, indicating enhanced digestive capacity on this tissue type. Conversely, senescent leaves consistently were associated with the lowest enzyme activities, with proteases (42.13 ± 1.37 × 10^−3^ units), amylases (33.00 ± 1.78 × 10^−3^ units), and lipases (1.01 ± 1.87 × 10^−3^ units) declining substantially, reflecting the reduced nutritional quality of this tissue. Intermediate enzyme levels were observed in nymphs fed young leaves and inflorescences, with protease (82.26 ± 1.28 and 78.47 ± 1.46 × 10^−3^ units) and lipase (2.21 ± 1.14 and 2.02 ± 1.33 × 10^−3^ units) activities exhibiting statistically comparable values between these two groups (DMRT; *p* > 0.05). Amylase activity, however, was significantly higher on young leaves (77.32 ± 1.54 × 10^−3^ units) than on inflorescences (63.43 ± 1.76 × 10^−3^ units). These findings demonstrate that the highest digestive enzyme activities were observed in nymphs fed mature leaves, whereas senescent tissues were associated with the lowest enzymatic activity, likely due to reduced nutritional quality and increased structural recalcitrance.

#### 3.3.2. Digestive Enzyme Profile of *D. chrysippus*

Quantitative assessment of digestive enzyme activities in fifth-instar larvae of *D. chrysippus* fed on different host parts of *C. gigantea* revealed a distinct allocation pattern compared to that observed in *P. pictus* (Table 6). Maximal enzymatic activities were consistently recorded on the young leaf diet, yielding the highest values for proteases (93.10 ± 1.56 × 10^−3^ units) and lipases (2.92 ± 1.23 × 10^−3^ units), while amylase activity on young leaves (86.20 ± 1.34 × 10^−3^ units) was statistically comparable to that of the inflorescence cohort (73.50 ± 1.37 × 10^−3^ units; DMRT, *p* > 0.05). Inflorescences supported moderate enzyme levels, with protease (71.40 ± 1.14 × 10^−3^ units) and lipase (2.00 ± 1.45 × 10^−3^ units) activities ranking second after young leaves, whereas mature leaves were associated with substantially lower activities for all three enzymes (proteases: 62.80 ± 1.14; amylases: 51.00 ± 1.57; lipases: 1.56 ± 1.51 × 10^−3^ units). Consistent with observations in *P. pictus*, senescent leaves induced the poorest enzymatic response, with protease (33.30 ± 1.78 × 10^−3^ units), amylase (38.10 ± 1.87 × 10^−3^ units), and lipase (0.32 ± 1.50 × 10^−3^ units) activities declining to their lowest levels. These findings indicate that the highest digestive enzyme activities in *D. chrysippus* were observed in larvae fed young leaves, while senescent tissues corresponded to the lowest enzymatic output, likely reflecting diminished nutritional availability and increased structural barriers to digestion.

### 3.4. Host Plant Biochemical Profiles

Biochemical characterization of *C. gigantea* tissues revealed substantial variation in nutritional and chemical composition across different plant structures (Table 7). Mature leaves exhibited peak concentrations of protein (125.27 ± 1.98 mg/g), lipids (38.25 ± 0.97 mg/g), nitrogen (2.38 ± 0.32%), phosphorus (0.38 ± 0.007%), and potassium (1.36 ± 0.05%), a pattern consistent with the dietary preferences of *P. pictus*, for which the highest digestive enzyme activities were recorded on this tissue. In contrast, young leaves were characterized by maximal carbohydrate content (125.42 ± 1.22 mg/g), water content (81 ± 3.15%), and water-to-nitrogen ratio (60.45%), parameters that collectively optimize nutritional conditions for *D. chrysippus* larvae, which performed best on this diet. The inflorescence served as a nutritionally balanced alternative, featuring the lowest fiber content (3.02 ± 0.97%) and intermediate levels of most macronutrients, including protein (122.13 ± 1.78 mg/g), carbohydrates (117.54 ± 1.09 mg/g), and lipids (33.22 ± 1.23 mg/g). Conversely, senescent leaves represented a severely degraded food source, exhibiting the lowest water content (39 ± 1.61%), protein (101.31 ± 1.11 mg/g), carbohydrates (98.07 ± 1.45 mg/g), lipids (21.14 ± 1.08 mg/g), nitrogen (1.03 ± 0.28%), phosphorus (0.24 ± 0.003%), and potassium (1.28 ± 0.07%). Critically, senescent leaves displayed elevated physical and chemical defense barriers, reaching maximal concentrations of total amino acids (0.830 ± 0.003 mg/g), phenolics (29.32 ± 1.42 mg/g), structural fiber (17.43 ± 0.74%), and toxic cardiac glycosides (302.81 ± 2.48 μg/100 mg), collectively rendering them nutritionally inferior and chemically deterrent to both insect species.

### 3.5. Free Fatty Acid Analysis

The free fatty acid (FFA) profile of *C. gigantea* shows highly distinct variations across tissue classes, peaking massively in mature leaves (95.6% total wt) and declining through inflorescences (76.43%) and senescent leaves (54.60%), down to a minimal footprint in young leaves (10.7%). Linoleic acid (C18:2) and Oleic acid (C18:1 d9) dominate the lipid landscape, reaching maximum concentrations in mature leaves at 36.3% and 30.3% respectively, while dropping significantly to just 4.1% and 6.6% in young tissue. Saturated fats like Palmitic (C16:0) and Stearic (C18:0) acids follow an identical trend, hitting zero in young leaves (Table 8). This stark lipid abundance in mature foliage explains why the mass-consuming orthopteran *P. pictus* achieves its absolute peak efficiency and tissue development on mature structures, successfully extracting dense fatty acid matrices unavailable in younger vegetative tissues.

### 3.6. Amino Acid Profiling of C. gigantea

The amino acid architecture of *C. gigantea* shifts dynamically across its lifespan, establishing distinct nutritional zones (Table 9). Young leaves function as highly concentrated transport hubs, featuring a massive abundance of mobile nitrogen via Asparagine (77.51 μmoles/g) and Valine (20.00 μmoles/g). Mature leaves transition into metabolically balanced factories, marked by peak concentrations of Glutamic acid (29.98 μmoles/g) and Glutamine (29.40 μmoles/g), alongside the exclusive presence of structural Hydroxyproline (13.77 μmoles/g). In contrast, senescent tissues undergo severe catabolic decay, characterized by a complete depletion of Proline (0.00 μmoles/g) and a major accumulation of metabolic waste in the form of Urea (54.12 μmoles/g).

### 3.7. GC-MS Phytochemical Profiles of C. gigantea Extracts

Gas chromatography–mass spectrometry (GC-MS) analysis reveals distinct volatile and secondary metabolite distributions across the four developmental structures of *C. gigantea* (Table 10, Table 11, Table 12 and Table 13). The young leaves are characterized by high concentrations of the diterpene alcohol Phytol (32.36%), followed by 9,12,15-Octadecatrienoic acid, methyl ester (23.57%) and the antioxidant 7,9-Di-tert-butyl-1-oxaspiro(4,5)deca-6,9-diene-2,8-dione (11.29%) (Table 10). The mature leaves are dominated heavily by the trisaccharide sugar Melezitose (24.21%), alongside elevated levels of 7,9-Di-tert-butyl-1-oxaspiro(4,5)deca-6,9-diene-2,8-dione (17.98%) and a reduced fraction of Phytol (16.49%) (Table 11). The senescent leaves exhibit an accumulation of 7,9-Di-tert-butyl-1-oxaspiro(4,5)deca-6,9-diene-2,8-dione (22.76%), a moderate retention of Phytol (18.16%), and prominent peaks of antimicrobial 1-Cyclohexyldimethylsilyloxy-3,5-dimethylbenzene (9.80%) and 2,4-Di-tert-butylphenol (7.66%) (Table 12). The inflorescence displays a highly unique chemical profile where 1,3-Propanediol, 2-ethyl-2-(hydroxymethyl)- functions as the major constituent (52.08%). Other notable components include 7,9-Di-tert-butyl-1-oxaspiro(4,5)deca-6,9-diene-2,8-dione (5.74%), 1,4-Benzenedicarboxylic acid, bis(2-ethylhexyl) ester (4.25%), and 9-Octadecenoic acid (Z)-, methyl ester (4.00%) (Table 13).

## 4. Discussion

### 4.1. Nutritional Ecology, Life-History Strategies, and Resource Partitioning

Herbivorous insects are continually confronted with the challenge of navigating spatial and temporal variations in the nutritional and defensive chemistry of their host plants. To optimize nutrient intake while minimizing or avoiding the ingestion of deleterious plant secondary metabolites, phytophagous insects rely on a suite of behavioral, physiological, and biochemical adaptations. These mechanisms include selective feeding on tissues of superior nutritional quality, mixing diets, or completely rejecting host tissues that contain lethal thresholds of toxic allelochemicals [12,13,14,15,16,17,18].

In the present investigation, the comparative dietary utilization, feeding behavior, and reproductive programming of the monarch butterfly *D. chrysippus* (Linn.) and the painted milkweed grasshopper *P. pictus* (Fab.) on *C. gigantea* reveal distinct physiological and ecological trade-offs. These trade-offs underscore fundamental differences between holometabolous and hemimetabolous life-history strategies when exploiting an exceptionally well-defended, cardenolide-rich host plant [11,19,20,21,22,23,24,25].

When a primary food resource becomes nutritionally inadequate, herbivorous insects generally employ compensatory feeding strategies, often accelerating consumption rates to satisfy baseline metabolic requirements for critical macroelements like nitrogen [26]. The efficacy and expression of this compensatory behavior vary substantially according to the herbivore’s degree of dietary specialization and the specific biochemical composition of the host tissue. In general, lepidopteran larvae exhibit a robust capacity to compensate for suboptimal nutritional density by increasing absolute ingestion rates. A clear manifestation of this compensatory feedback loop was observed in *D. chrysippus* larvae when restricted to young leaves. The absolute growth rate (GR) and post-embryonic development of lepidopteran larvae are tightly coupled to the volumetric moisture content of their food [27,28,29,30,31].

The significantly depressed growth rates observed on low-moisture diets stem from a reduction in the efficiency of converting assimilated matter into larval biomass (ECD), rather than a failure to ingest or digest the material. Conversely, *P. pictus* nymphs demonstrate a markedly different physiological response to moisture. For this orthopteran, an excess of dietary water poses a distinct physiological challenge, disrupting homeostatic digestive mechanics, depressing absolute consumption, and subsequently hindering growth. This divergence highlights a fundamental difference in water regulation: *D. chrysippus* requires highly hydrated tissues to sustain its rapid, biomass-accumulating larval phase, whereas *P. pictus* thrives on more fibrous, moderately hydrated foliage.

The intensity and temporal dynamics of feeding activity were significantly higher in *D. chrysippus* than in *P. pictus*. In holometabolous insects, the feeding environment of early-instar larvae is largely predetermined by the adult female’s oviposition site selection [32]. Nutritional indices provide a valuable framework for assessing host tissue quality, though they must be interpreted alongside the defensive chemical profile of the plant. In this study, *D. chrysippus* achieved peak nutritional efficiency and maximum food utilization when fed young leaves of *C. gigantea*. In contrast, *P. pictus* exhibited optimal food utilization on mature leaves. On the whole, *C. gigantea* foliage was converted to body mass with higher overall efficiency by *D. chrysippus* than by *P. pictus*. This disparity reflects the intense energetic demands of holometabolous development, where larval stages must accumulate vast lipid and protein reserves to fuel the non-feeding pupal stage and subsequent metamorphosis.

Significant shifts in nutritional indices frequently reflect the metabolic costs of processing plant allelochemicals or adapting to structural leaf modifications [10,33,34]. In our experiments, *D. chrysippus* systematically avoided the elevated cardiac glycoside (cardenolide) concentrations characteristic of mature and senescent *C. gigantea* leaves. For *P. pictus*, however, the mature leaves presented an optimal chemical and physical matrix. The primary determinants of foliar quality for insect herbivores are widely understood to include nitrogen concentration, moisture content, allelochemical profiles, and physical barriers such as tissue toughness and pubescence [11,35,36,37]. Relative growth rates (RGR) correlate strongly with foliar water and nitrogen content [29]. Because most phytophagous insects derive their moisture directly from their food, water intake must be regulated either by selecting tissues with specific moisture profiles or by adjusting consumption rates to meet physiological needs [38]. Low foliar water content remains a primary indicator of poor nutritional quality in plant tissues [36].

In this investigation, mature *C. gigantea* leaves contained higher total nitrogen concentrations than either young or senescent leaves. Despite this, *D. chrysippus* consistently preferred young leaves, which supported higher consumption rates and elevated relative growth rates (RGR). This pattern aligns with observations by Scriber [27] in *Hyalophora cecropia*, where low tissue moisture degraded food conversion efficiencies. Furthermore, an extensive review of 25 lepidopteran species by Slansky and Scriber [11] demonstrated that maximum values for dry mass digestion and absorption decline sharply when both foliar water and nitrogen are limited. For lepidopterans, water acts as a critical limiting factor for growth and development, whereas orthopterans show greater tolerance for, and adaptation to, lower moisture regimes.

### 4.2. Physiological Efficiencies and Metabolic Costs

The efficiency of conversion of digested food (ECD) measures an insect’s capacity to allocate assimilated nutrients toward somatic growth. Consequently, a drop in ECD indicates that a higher proportion of energy is being diverted away from biomass accumulation to cover increased metabolic maintenance and detoxification costs [11]. In the present study, *D. chrysippus* larvae fed young leaves exhibited optimal consumption rates, accelerated growth, and high nutritional efficiencies. Conversely, *P. pictus* avoided these highly hydrated young leaves. Excessive foliar moisture imposes severe volumetric constraints on grasshoppers, over-inflating the crop and midgut, which limits physical intake capacity and slows digestive processing [39,40,41,42,43,44,45]. For orthopterans, feeding on tissues with lower moisture levels often optimizes growth, survival, and fecundity.

A lower level of body tissue hydration increases the efficiency of water utilization for growth by enabling the hydration of additional new biomass per unit of absorbed water [46]. Because mature *C. gigantea* leaves feature high nitrogen concentrations paired with lower moisture levels and intermediate cardenolide profiles, *P. pictus* nymphs reared on this diet achieved their peak physiological performance. The water-to-nitrogen ratio in mature leaves appears uniquely optimized for orthopteran nymphal development. Dietary nitrogen exerts a profound regulatory influence on consumption, assimilation, and overall food utilization in insects [26,36]. The distinct reduction in consumption and weight gain observed when *D. chrysippus* was restricted to mature leaves can be attributed to the combined effects of reduced moisture, higher nitrogen density, and elevated cardenolide concentrations.

The high water-to-nitrogen ratio and low cardenolide concentrations found in young leaves make them exceptionally suitable for *D. chrysippus*, optimizing both intake and approximate digestibility (AD). For *P. pictus*, however, the lower moisture, higher nitrogen, and intermediate cardenolide concentrations characteristic of mature leaves stimulated both consumption and food utilization efficiency. These results demonstrate that the interactive effects of the foliar water-nitrogen index and cardenolide concentration serve as primary drivers of growth and dietary utilization for both species [47,48].

Insects feeding on protein- and nitrogen-rich plant tissues routinely achieve greater developmental success than those restricted to nutrient-depleted foliage [48,49]. In our experiments, *P. pictus* exhibited maximal survival and development on mature leaves, while *D. chrysippus* peaked on young leaves, directly tracking the higher digestible protein concentrations in these respective tissues relative to senescent foliage. Senescent *C. gigantea* leaves displayed severe nutritional depletion, characterized by reduced total carbohydrates and degraded proteins. According to Swain [50], primary nutrients like proteins and structural carbohydrates become physically locked away in senescent or aging leaves due to extensive hydrogen bonding with lignins and structural polyphenols, rendering them largely inaccessible to insect digestive enzymes.

### 4.3. Mechanisms of Allelochemical Impact and Detoxification

Insect feeding behavior is peripherally regulated by a delicate sensory balance between phagostimulatory nutrients and deterrent allelochemicals [51]. Secondary plant metabolites can function as potent feeding stimulants or severe deterrents depending entirely on the insect’s evolutionary specialization. In this study, senescent leaves contained the highest concentrations of cardenolides, which significantly disrupted food utilization in both *P. pictus* and *D. chrysippus*. When either species was fed senescent foliage, approximate digestibility (AD) and the efficiency of conversion of digested food (ECD) dropped significantly. This physiological decline reflects the energetic costs of dealing with high cardenolide concentrations. However, AD should not be interpreted as a direct measure of true nutrient digestibility. As a gravimetric index, AD measures the net disappearance of dry matter from the gut but does not distinguish between material actually assimilated across the midgut epithelium and material simply retained for prolonged periods [10,11]. Several factors can confound AD values independently of true assimilation efficiency. First, gut transit time substantially affects AD: rapid passage may yield low AD due to insufficient digestion time, whereas extended retention can artificially inflate AD by prolonging enzyme exposure without conferring nutritional benefit [10,11]. Second, indigestible dietary fiber and food bulk cause a dilution effect that reduces the relative contribution of digestible fractions [10]. Third, plant secondary metabolites, particularly cardenolides and phenolics, can interfere with gut epithelial transport or bind dietary proteins, reducing net absorption while leaving gross dry matter disappearance apparently unchanged [1]. These confounding factors are particularly relevant to the present dataset. Notably, *D. chrysippus* larvae feeding on senescent *C. gigantea* leaves exhibited relatively high AD values (54.19%) alongside severely depressed growth rates and reduced conversion efficiencies. This apparent paradox suggests that elevated AD on senescent foliage may reflect prolonged gut retention—a physiological response to nutrient scarcity—rather than genuine high assimilation [10,11]. This interpretation is supported by the concurrent decline in ECD on the same diet, indicating that even if material is apparently “digested,” post-assimilatory conversion remains poor due to detoxification costs [3]. Therefore, AD should be interpreted cautiously and in conjunction with other nutritional indices (ECI, ECD, RGR) rather than as a standalone measure of dietary quality [10,11]. Historically, the protein-binding properties of plant phenolics and tannins were thought to universally reduce dietary protein availability [52] and inactivate digestive enzymes [50]. While this generalization has been refined by the discovery of specialized gut surfactants that prevent phenolic-protein complexation in many species [53], senescent foliage presents a combined challenge of complex phenolics and high cardenolide concentrations that disrupts standard digestive physiology. Interestingly, both *P. pictus* nymphs and *D. chrysippus* larvae excreted significantly fewer fecal pellets when restricted to senescent leaves. This decrease indicates a substantial slowing of gut motility, possibly representing a physiological attempt to maximize the extraction of scarce nutrients from poor-quality food, or reflecting a direct disruptive effect of cardenolides on the semi-peristaltic coordination of the insect alimentary canal. The lower relative growth rates (RGR) associated with a senescent leaf diet are primarily due to allelochemical-induced feeding deterrency. Furthermore, the low ECD values recorded on senescent leaves indicate elevated catabolic and excretory costs associated with metabolic detoxification [11,54].

The simultaneous decline in consumption, growth, and conversion efficiencies on senescent foliage highlights the combined impacts of structural toughness, fiber content, and altered chemistry [54,55,56], driven by depleted nutrients, accumulated phenolics, and low moisture [57,58]. As noted by Slansky [59], variations in foliar water and fiber content directly alter nutritional indices.

Digestibility can also be compromised by post-absorptive allelochemical interference within the midgut tissue, rendering the diet poorly suited for growth, development, or long-term survival [60]. High fiber levels in senescent leaves limit protein availability, driving both species to actively select the nutrient-dense, less fibrous alternative tissues found in young and mature foliage. Because mature *C. gigantea* leaves present a rich nutrient profile alongside an intermediate cardenolide concentration, *P. pictus* nymphs actively select this tissue. By feeding on mature foliage, this specialized grasshopper can safely process, sequester, or metabolically convert absorbed allelochemicals into alternative active forms for its own chemical defense. The chemical modification of absorbed host plant allelochemicals into defensive analogs, along with the utilization of primary nutrients to maintain sequestration mechanisms, is a well-documented evolutionary strategy among chemically defended aposematic insects. *P. pictus* exemplifies this adaptation, utilizing the complex chemical profile of mature *C. gigantea* leaves to support both its nutritional ecology and its defense systems.

While plant phenols are widely recognized as feeding deterrents for generalist phytophagous insects [61], certain specialized species can utilize them effectively. For example, the tree locust *Anacridium melanorhodon* exhibits enhanced survival and faster growth when specific plant phenols are incorporated into diets low in protein, utilizing tannic acid to stabilize cuticular proteins [62]. In the case of *P. pictus*, the distinct cardenolide profile of mature *C. gigantea* leaves appears to play a similar supportive role, actively influencing growth and developmental coordination. The heavily sclerotized exoskeleton of insects constitutes a large fraction of total dry body mass and places significant demands on phenolic and amino acid pools during molting [63]. Enhanced growth has been observed in *Bombyx mori* larvae when phenolic acids, such as gallic or protocatechuic acid, are added to artificial diets [64], and phenolics have been shown to stimulate feeding in several specialized herbivores [65].

However, catechol-based phenolics like chlorogenic acid and rutin can also act as deterrents, reducing amino acid bioavailability and non-specifically inhibiting digestive enzymes in non-adapted species [66,67]. In senescent *C. gigantea* leaves, the combination of high cardenolide concentrations and accumulated phenolics appears to impair nutritional efficiency in both *D. chrysippus* and *P. pictus*, likely by interfering with amino acid absorption and metabolic processing. The critical role of dietary amino acid composition in supporting insect growth and reproductive success is well established [68,69]. Total free amino acid concentrations in host plants are highly dynamic and vary with tissue age. Our biochemical profiling of *C. gigantea* foliage revealed significantly higher total free amino acid concentrations in young and mature leaves compared to senescent tissues. This variation directly correlates with the superior growth, accelerated development, and enhanced reproductive performance observed when *D. chrysippus* and *P. pictus* were reared on their respective preferred leaf stages.

### 4.4. Plant Architecture, Physical Barriers, and Digestive Physiology

The decline in AD on senescent foliage also reflects the high proportion of indigestible structural components in aging tissues. This pattern aligns with findings by Mukerji and Guppy [70], Kogan and Cope [71], and Adler and Grebenok [72], which demonstrate that decreases in AD are often tied to the consumption of highly lignified structural materials. Because AD measures the net proportion of ingested food absorbed across the midgut epithelium, any allelochemical or structural barrier that disrupts cellular transport or binds nutrients will lower this index. Similarly, ECD falls when absorbed allelochemicals exert toxic effects that demand metabolic energy for detoxification, diverting resources away from tissue synthesis.

Physical attributes, particularly tissue toughness driven by advanced lignification and elevated cellulose content, present a major obstacle to insect feeding. Senescent *C. gigantea* leaves exhibited significantly higher tissue resistance and toughness than young or mature leaves. This increased structural defense clearly deterred feeding in both *D. chrysippus* larvae and *P. pictus* nymphs. Tough foliage has been shown to wear down the cutting margins of insect mandibles, as observed in *Plagiodera versicolora*, leading to reduced ingestion rates and a subsequent drop in fecundity [73,74]. Diets high in indigestible bulk, fiber, and lignified tissue are generally associated with reduced foliar moisture, lower total protein, and elevated cardenolide concentrations [26,75]. Structural leaf fibers like lignin and cellulose are primary drivers of the lower AD values observed on senescent foliage, where aging processes alter the plant’s overall chemical and physical profile. Cardenolides are exceptionally abundant secondary metabolites in milkweeds, frequently causing strong feeding deterrency or severe growth inhibition in non-adapted or stressed herbivores [76,77].

The digestive enzyme systems of phytophagous insects are typically fine-tuned to match the specific nutritional composition of their preferred host tissues [78]. The balanced mix of proteins, soluble carbohydrates, and high moisture in young leaves was associated with both digestion and conversion efficiencies (ECI and ECD) in *D. chrysippus*. In this study, the activities of key digestive enzymes—including midgut proteases and carbohydrases—were highest in *P. pictus* nymphs and *D. chrysippus* larvae fed mature and young leaves, respectively. This response aligns with patterns documented by Baker [79], where coleopteran larvae feeding on protein-rich diets exhibited elevated protease activity and reduced amylase activity compared to species feeding on carbohydrate-heavy grains. Furthermore, feeding activity itself has been reported to correlate with enzyme synthesis and secretion within the insect gut [80,81,82,83,84]. As noted by Chapman [85], total enzyme production is closely tied to feeding behavior and the volume of food passing through the alimentary canal.

Senescent *C. gigantea* foliage clearly disrupted both the dietary utilization patterns and the underlying digestive physiology of *P. pictus* and *D. chrysippus*. Shifts in the activity or expression of core digestive enzymes can alter an insect’s entire metabolic network [86,87]. Because feeding performance directly influences every stage of insect growth and reproductive potential, these enzyme disruptions have broad biological impacts [11,31]. In our experiments, both *P. pictus* and *D. chrysippus* displayed shorter gut transit times and increased fecal pellet egestion rates when feeding on young leaves compared to older foliage. This faster passage indicates smooth movement of the food bolus through the alimentary canal, matching elevated digestive enzyme levels and high ingestion efficiencies. Foliar water and nitrogen content appear to be key drivers of this efficient gut clearance. High concentrations of protein, nitrogen, moisture, and essential minerals in young and mature leaves likely provide the physiological stimulation needed to trigger secretor cells within the midgut epithelium.

### 4.5. Macronutrient Allocation and Reproductive Programming

For the vast majority of insects studied, dietary carbohydrates are essential for fueling metabolic activity and supporting optimal growth rates [88]. While insects can utilize lipids and amino acids as alternative energy substrates, they consistently exhibit higher vitality and survival rates when provided with adequate dietary carbohydrates [89]. As House [90] observed, carbohydrate deficiency typically manifests as a systemic loss of vitality and physical activity rather than a localized defect. When restricted to senescent leaves, both species showed reduced growth and development. This loss of metabolic vitality led to shortened adult lifespans and a sharp decline in lifetime fecundity, a pattern also seen in other insects under nutritional stress [91]. Dietary lipids play equally critical and varied roles in insect biology. They serve as the primary fuel source during periods of fasting, diapause [92], extended migratory flights [93], and non-feeding pupal metamorphosis [92,94,95].

Research in lepidopterans like *Pieris brassicae* and various noctuids indicates that foliar glycolipids and phospholipids are rapidly hydrolyzed within the midgut, serving as the primary source of essential fatty acids [96,97,98]. These polar leaf lipids represent a key nutritional requirement for phytophagous larvae. Furthermore, lipids are strictly required by all insects because they cannot synthesize sterols pathways from scratch. Sterols are essential structural components of cellular membranes, aid in lipoprotein transport, and serve as precursors for ecdysteroids, the insect molting hormones [99]. Compared to proteins and carbohydrates, total lipid concentrations were extremely low in senescent *C. gigantea* leaves. The frequent molting failures and high mortality observed in early-instar insects fed senescent foliage can be attributed to this severe lipid deficiency, compounded by low tissue moisture.

Insects utilizing protein-rich host plants consistently achieve greater reproductive and developmental success than those feeding on protein-depleted tissues [48,49]. In the current study, *P. pictus* and *D. chrysippus* achieved maximum fecundity and survival when reared on mature leaves, young leaves, or inflorescences, tracking the higher digestible protein concentrations in these preferred tissues relative to senescent foliage. Absorbed dietary proteins are allocated by adult females directly toward metabolic maintenance and oogenesis [100,101]. In many species, the development of the female reproductive system depends heavily on nutrient reserves accumulated during larval feeding, supplemented by adult dietary intake [102]. When fed young or mature leaves, both *P. pictus* and *D. chrysippus* were able to allocate substantial energy reserves toward egg production, resulting in elevated reproductive indices. As summarized by Slansky and Scriber [11], an insect’s reproductive timing, egg production rate, and egg quality depend closely on the total nutrients accumulated during the larval feeding phase, balanced alongside the availability and quality of adult food.

The nutritional state of the female directly determines vitellogenin synthesis and yolk deposition levels [103]. Under nutritional stress or low food availability, females frequently produce smaller egg pods or lay eggs with reduced yolk reserves [103,104]. Reduced food consumption thus leads to extended developmental times, smaller adult body sizes, and a significant drop in lifetime fecundity [11,26,103,105,106]. Decreased adult longevity and reduced fecundity were highly pronounced in insects reared on senescent foliage, driven by low nutrient availability and high concentrations of phenolics and cardenolides. Both species exhibited suboptimal growth and poor food utilization on senescent leaves, a response tied to a natural deficiency of critical precursor amino acids like phenylalanine and tyrosine, alongside the complete absence of phosphoethanolamine, hydroxyproline, alanine, proline, amino-n-butyric acid, and hydroxylysine.

Work by Bernays and Woodhead [62] showed that dietary supplementation with phenylalanine significantly boosts nutritional efficiency indices in the desert locust *Schistocerca gregaria*. Furthermore, senescent leaves contained high concentrations of metabolic urea compared to other leaf stages, which likely contributed to the depressed growth and developmental abnormalities observed in senescent-reared cohorts. As noted by Schow-Madsen et al. [107] and Lee, [108], even essential amino acids can prove harmful if ingested in excessive amounts or if they are not properly balanced with other primary amino acids.

The complete depletion of proline in senescent *C. gigantea* leaves (0.00 μmoles/g) relative to young (22.56 μmoles/g) and mature (26.88 μmoles/g) tissues is of particular significance for insect physiology. Proline serves multiple critical functions: (i) it acts as a major constituent of structural proteins, particularly cuticular proteins where proline hydroxylation to hydroxyproline stabilizes collagen-like triple helical domains; (ii) it functions as an osmoprotectant and energy substrate during periods of physiological stress; and (iii) it serves as a precursor for glutamine synthesis, which is essential for nitrogen metabolism and ammonia detoxification [85]. The absence of proline in senescent foliage likely contributes to the observed reduction in ECD values (*P. pictus*: 48.74% vs. 54.32% on young leaves; *D. chrysippus*: 27.48% vs. 50.22% on young leaves), indicating that assimilated nutrients are being diverted away from somatic growth toward meeting elevated metabolic maintenance costs.

The concurrent accumulation of urea in senescent leaves (54.12 μmoles/g) compared to young (18.35 μmoles/g) and mature (32.12 μmoles/g) tissues represents a further metabolic burden. Elevated dietary urea imposes direct physiological costs on herbivorous insects, as it must be eliminated via the excretory system (typically as uric acid in Lepidoptera or ammonia in Orthoptera), consuming energy and diverting nitrogen from anabolic pathways. High urea levels in senescent foliage may also reflect protein degradation and amino acid deamination during leaf senescence, resulting in the accumulation of nitrogenous waste products that are nutritionally unavailable or even physiologically detrimental to insects. This nitrogenous waste burden likely contributes to the reduced larval growth rates and extended developmental durations observed in both species when reared on senescent foliage.

The absence of phosphoethanolamine (0.00 μmoles/g in senescent vs. 0.188 μmoles/g in mature leaves) and hydroxylysine (0.00 μmoles/g in senescent vs. 13.77 μmoles/g in mature leaves) further compromises insect development. Phosphoethanolamine is a precursor for phosphatidylethanolamine, a major phospholipid component of cell membranes and essential for cellular integrity and function. Hydroxylysine is required for the stabilization of collagen-like cuticular proteins and plays a critical role in the structural integrity of the insect exoskeleton. The absence of these two amino acids in senescent tissue likely contributes to the increased mortality and molting failures observed in senescent-reared cohorts, particularly during ecdysis when cuticular protein synthesis is maximized.

Collectively, these amino acid profile shifts (proline depletion, urea accumulation, and the absence of phosphoethanolamine and hydroxylysine) create a nutritionally deficient and metabolically stressful dietary environment that manifests as reduced digestive enzyme activity, impaired nutrient assimilation, and compromised developmental fitness. The 45–68% reduction in protease and amylase activities observed in senescent-reared insects (Table 5 and Table 6) may be directly linked to the altered amino acid milieu, as enzyme synthesis and turnover are regulated by amino acid availability and nitrogen balance.

### 4.6. Evolutionary Adaptations and Specialized Feeding Behaviors

Senescent *C. gigantea* leaves combine low moisture, poor nutrition, and high cardenolide levels, deterring both insect species [26,75]. Phytophagous insects face the ongoing challenge of selecting not only the right host plant species within a community, but also the optimal tissue types on an individual plant. Insects typically exhibit strong preferences for specific host tissues that maximize reproductive success, whether for the ovipositing adult or the developing larvae [109,110,111,112,113,114,115]. The insect must ingest, digest, and assimilate essential nutrients, converting them into the energy and structural components required for survival, growth, and reproduction.

The most preferred plant tissues are those that meet these dietary requirements while being easily digested and metabolized [11]. In this investigation, both *D. chrysippus* and *P. pictus* showed significant differences in dietary utilization across the various leaf age classes (young, mature, senescent leaves, and inflorescences) of *C. gigantea*. Food utilization peaked when *P. pictus* was reared on mature leaves. These variations in consumption and assimilation directly shaped the reproductive output of adult females. Maximum egg production occurred when *D. chrysippus* was reared on young leaves and *P. pictus* on mature leaves. These preferred diets also supported the highest growth rates and shortest development times for each species. Insects exploiting different host plants or tissues display variations in assimilation efficiency that reflect both leaf chemistry and moisture content [11]. When *D. chrysippus* larvae and *P. pictus* nymphs were restricted to senescent leaves, their approximate digestibility (AD) values fell to their lowest levels.

To overcome the potent latex defenses of their host, both species have evolved specialized behavioral adaptations. *P. pictus* nymphs were observed to consume leaves starting from the margins, moving systematically from tip to base, and making deliberate incisions along the abaxial surface of the midrib. This behavior effectively cuts off and drains the latex prior to feeding. Similarly, *D. chrysippus* larvae systematically sever the primary leaf veins before chewing on the lamina. This vein-cutting behavior is a classic adaptation among insect herbivores specialized on milkweeds, allowing them to circumvent the plant’s high-pressure laticifer system [116,117,118,119,120,121]. The two species differ in their cutting tactics. *P. pictus* makes several cuts along the underside of the midrib, whereas *D. chrysippus* severs the main vein with a single precise cut from above. The grasshopper’s repeated cuts appear to gradually release latex pressure from the leaf tip downward; the butterfly’s single cut isolates the feeding site by blocking latex flow from both directions [119]. These differences may reflect the distinct developmental constraints of each species. As a hemimetabolous insect with gradual mandibular growth, *P. pictus* employs a stepwise approach that suits its prolonged feeding across multiple instars. The holometabolous *D. chrysippus*, in contrast, must accumulate resources within a short larval period and benefits from a rapid, one-cut strategy that maximizes feeding efficiency [118]. The divergence also parallels their dietary preferences: *P. pictus* favors mature leaves with intermediate cardenolide levels, while *D. chrysippus* prefers young leaves with lower cardenolide concentrations.

In studies of the related monarch *Danaus plexippus*, Zalucki et al. [122], and Oyeyele and Zalucki [123] found that female oviposition strongly favors plants with intermediate cardenolide levels. In our study, *P. pictus* preferred mature leaves (which feature intermediate cardenolide concentrations), while *D. chrysippus* maximized efficiency on young leaves (low cardenolide concentrations). This pattern reflects the clear physiological costs that individual herbivores incur when processing host cardenolides [124].

Zalucki and Brower [125] analyzed the survival of *D. plexippus* on *Asclepias humistrata* across multiple locations, demonstrating that larval mortality correlates with increasing cardenolide levels and advancing plant age. We observed a similar decline in longevity and survival when *P. pictus* and *D. chrysippus* were reared on senescent *C. gigantea* leaves, which carry high cardenolide concentrations. This reduced growth and survival stem from the combination of high cardenolide toxicity and increased tissue toughness in senescent leaves [119]. Zalucki and Brower [125] concluded that the latex system serves as a primary defense mechanism in milkweeds, remaining highly effective even against specialized herbivores. Moreover, milkweeds have been extensively analyzed for their chemical profiles, driven in part by interest in their potential as sources of industrial rubber and specialized oils [126]. They contain a complex array of compounds, including isoprene derivatives (α- and β-amyrin and their respective acetates) [116], with substantial variation in cardenolide profiles across different plant species [127]. Furthermore, total cardenolide levels change seasonally and vary significantly among individual leaves on the same plant [125,128,129]. In the present study, the shifting cardenolide profiles across different leaf ages and inflorescences of *C. gigantea* exerted a powerful regulatory influence, driving clear physiological and ecological trade-offs in both *P. pictus* and *D. chrysippus*.

## 5. Conclusions

This study concludes that the tri-trophic interaction between *C. gigantea* and its specialized herbivores is heavily dictated by age-dependent variations in foliar chemistry, moisture, and structural defense profiles. The holometabolous butterfly, *D. chrysippus*, exhibits a strong preference for highly hydrated, nutrient-dense young leaves, which optimize food conversion efficiencies (ECD) to fuel rapid larval development and metamorphosis. Conversely, excessive water poses a physiological challenge to the hemimetabolous grasshopper, *P. pictus*; this species reaches peak physiological performance and maximized egg production on fibrous mature leaves, which feature an optimal, well-balanced water-to-nitrogen ratio. Both species exhibit compromised growth, depleted digestive enzyme activity, and diminished reproductive success when restricted to senescent leaves. Collectively, these profiles demonstrate a clear nutritional divergence between the two herbivores sharing the same host plant. While the hemimetabolous *P. pictus* optimizes its digestive physiology to target mature leaves, the holometabolous *D. chrysippus* adapts its enzyme upregulation toward highly hydrated, nutrient-dense young leaves to maximize assimilation prior to pupation. This poor performance stems from a combination of advanced tissue toughness, structural fiber barriers, severe macronutrient/amino acid depletion, and toxic cardenolide accumulation. To overcome the plant’s high-pressure laticifer defenses, both species employ distinct, sophisticated behavioral adaptations—such as vein-cutting by *D. chrysippus* larvae and abaxial midrib incisions by *P. pictus* nymphs—to block and drain toxic latex prior to feeding. Ultimately, host plant tissue suitability serves as a primary driver shaping the evolutionary trade-offs, resource partitioning, and reproductive fitness of these co-occurring insects.

## Figures and Tables

**Table 1:** Biological and reproductive parameters of *Poecilocerus pictus* fed on different plant parts of wild giant milkweed *Calotropis gigantea*.

Biological Parameters	Host Parts			
	Inflorescence	Young Leaves	Mature Leaves	Senescent Leaves
I Instar	12.10 ± 1.86 ^b^	11.20 ± 1.67 ^a^	10.00 ± 1.89 ^a^	12.40 ± 1.36 ^b^
II Instar	11.54 ± 1.37 ^b^	10.36 ± 1.89 ^a^	9.59 ± 1.56 ^a^	12.10 ± 1.67 ^b^
III Instar	12.20 ± 1.57 ^b^	10.90 ± 1.34 ^a^	10.72 ± 1.23 ^a^	12.96 ± 1.98 ^b^
IV Instar	12.45 ± 1.55 ^b^	11.40 ± 1.23 ^b^	8.40 ± 1.56 ^a^	13.17 ± 1.35 ^c^
V Instar	12.38 ± 1.23 ^b^	10.45 ± 1.34 ^a^	10.29 ± 1.78 ^a^	14.80 ± 1.67 ^c^
Total Nymphal Duration	56.57 ± 1.34 ^b^	55.51 ± 1.67 ^ab^	49.00 ± 1.12 ^a^	63.98 ± 1.87 ^c^
Pre-oviposition Period	25.38 ± 1.78 ^b^	22.50 ± 1.59 ^ab^	20.50 ± 1.34 ^a^	29.36 ± 1.89 ^c^
Oviposition Period	36.00 ± 1.89 ^b^	30.38 ± 1.23 ^c^	52.20 ± 1.47^d^	15.25 ± 1.75 ^a^
Post-oviposition Period	22.55 ± 1.47 ^b^	19.00 ± 1.46 ^b^	29.56 ± 1.81 ^c^	11.28 ± 1.25 ^a^
Longevity (Male)	95.35 ± 1.23 ^b^	104.55 ± 1.87 ^c^	116.23 ± 1.11 ^d^	88.76 ± 1.16 ^a^
Longevity (Female)	118.50 ± 1.34 ^b^	127.39 ± 1.92 ^c^	140.70 ± 1.00 ^d^	109.84 ± 1.18 ^a^
Fecundity (No. of eggs)	434.61 ± 1.56 ^b^	407.17 ± 1.00 ^b^	511.96 ± 1.12 ^c^	271.75 ± 1.79 ^a^

Note: Within a row, means followed by different lowercase letters are significantly different at the 5% level by DMRT (Duncan’s Multiple Range Test).

**Table 2:** Biological and reproductive parameters of *Danaus chrysippus* fed on different plant parts of wild giant milkweed *C. gigantea*.

Biological Parameters	Host Parts			
	Inflorescence	Young Leaves	Mature Leaves	Senescent Leaves
Total Larval Duration (days)	11.36 ± 1.23 ^a^	10.12 ± 1.67 ^a^	12.06 ± 1.89 ^a^	14.38 ± 1.54 ^b^
Prepupal Period (days)	2.86 ± 1.29 ^b^	2.26 ± 1.78 ^a^	2.64 ± 1.86 ^b^	3.22 ± 1.57 ^c^
Pupal Period (days)	10.44 ± 1.33 ^a^	9.50 ± 1.99 ^a^	10.48 ± 1.34 ^a^	11.54 ± 1.76 ^b^
Pre-oviposition Period (h)	3.69 ± 1.17 ^b^	3.11 ± 1.25 ^a^	3.55 ± 1.51 ^b^	4.16 ± 1.47 ^c^
Oviposition Period (h)	17.23 ± 1.57 ^c^	23.07 ± 1.67 ^d^	13.86 ± 1.57 ^b^	6.18 ± 1.54 ^a^
Post-oviposition Period (h)	4.81 ± 1.69 ^b^	5.00 ± 1.78 ^b^	4.35 ± 1.14 ^ab^	3.73 ± 1.14 ^a^
Fecundity (Total No. of eggs)	113.80 ± 1.33 ^c^	162.70 ± 1.46 ^d^	72.62 ± 1.47 ^b^	60.16 ± 1.89 ^a^
Male Adult Longevity (days)	8.34 ± 1.67 ^c^	9.56 ± 1.23 ^c^	5.34 ± 1.14 ^b^	3.38 ± 1.14 ^a^
Female Adult Longevity (days)	8.72 ± 1.23 ^b^	10.48 ± 1.69 ^c^	4.78 ± 1.23 ^a^	3.34 ± 1.37 ^a^

Note: Within a row, means followed by different lowercase letters are significantly different at the 5% level by DMRT (Duncan’s Multiple Range Test).

**Table 3:** Food utilization efficiency measures of the penultimate instars nymph of *P. pictus* fed on different aged leaves and inflorescence of wild giant milkweed *C. gigantea*.

Host Parts/Leaves	CI (g)	RGR (g)	AD (%)	ECI (%)	ECD (%)
Inflorescence	0.310 ± 0.018 ^b^	0.076 ± 0.005 ^b^	47.16 ± 2.34 ^a^	24.83 ± 1.56 ^a^	52.66 ± 2.18 ^c^
Young Leaves	0.315 ± 0.021 ^b^	0.077 ± 0.006 ^b^	45.15 ± 2.67 ^a^	24.52 ± 1.89 ^a^	54.32 ± 2.45 ^c^
Mature Leaves	0.271 ± 0.015 ^a^	0.064 ± 0.004 ^a^	55.96 ± 2.89 ^b^	23.86 ± 1.78 ^a^	42.62 ± 2.01 ^a^
Senescent Leaves	0.302 ± 0.019 ^b^	0.071 ± 0.005 ^ab^	48.91 ± 2.56 ^a^	23.83 ± 1.67 ^a^	48.74 ± 2.23 ^b^

Note: Within a column means followed by different lowercase letters are significantly different at 5% level by DMRT.

**Table 4:** Food utilization efficiencies of fifth instar *D. chrysippus* fed on different plant parts of wild giant milkweed *C. gigantea*.

Host Parts/Leaves	CI (g)	RGR (g)	AD (%)	ECI (%)	ECD (%)
Inflorescence	0.416 ± 0.023 ^a^	0.073 ± 0.005 ^b^	46.98 ± 2.45 ^b^	17.67 ± 1.34 ^b^	37.62 ± 1.89 ^b^
Young Leaves	0.458 ± 0.025 ^a^	0.110 ± 0.007 ^a^	48.15 ± 2.56 ^b^	24.18 ± 1.67 ^a^	50.22 ± 2.34 ^a^
Mature Leaves	0.351 ± 0.019 ^b^	0.053 ± 0.004 ^c^	45.31 ± 2.34 ^b^	15.35 ± 1.23 ^b^	33.88 ± 1.78 ^bc^
Senescent Leaves	0.280 ± 0.016 ^c^	0.041 ± 0.003 ^d^	54.19 ± 2.78 ^a^	14.89 ± 1.45 ^b^	27.48 ± 1.56 ^c^

Note: Within a column means followed by different lowercase letters are significantly different at 5% level by DMRT.

**Table 5:** Digestive enzyme profiles of V instar nymph’s of *P. pictus* fed on host parts of wild giant milkweed *C. gigantea*.

Enzymes	Young Leaves	Mature Leaves	Senescent Leaves	Inflorescence
Proteases	82.26 ± 1.28 ^b^	102.05 ± 1.57 ^c^	42.13 ± 1.37 ^a^	78.47 ± 1.46 ^b^
Amylases	77.32 ± 1.54 ^b^	99.98 ± 1.14 ^c^	33.00 ± 1.78 ^a^	63.43 ± 1.76 ^b^
Lipases	2.21 ± 1.14 ^b^	4.02 ± 1.00 ^c^	1.01 ± 1.87 ^a^	2.02 ± 1.33 ^b^

Note: Within a row, means followed by different lowercase letters are significantly different at the 5% level by DMRT.

**Table 6:** Digestive enzyme profiles of V instar larvae of *D. chrysippus* fed on host parts of wild giant milkweed *C. gigantea*.

Enzymes	Young Leaves	Mature Leaves	Senescent Leaves	Inflorescence
Proteases	93.1 ± 1.56 ^d^	62.8 ± 1.14 ^b^	33.3 ± 1.78 ^a^	71.4 ± 1.14 ^c^
Amylases	86.2 ± 1.34 ^c^	51.0 ± 1.57 ^b^	38.1 ± 1.87 ^a^	73.5 ± 1.37 ^c^
Lipases	2.92 ± 1.23 ^d^	1.56 ± 1.51 ^b^	0.32 ± 1.50 ^a^	2.00 ± 1.45 ^c^

Note: Within a row, means followed by different lowercase letters are significantly different at the 5% level by DMRT.

**Table 7:** Biochemical parameters of wild giant milkweed *C. gigantea*.

Biochemical Parameters	Young Leaves	Mature Leaves	Senescent Leaves	Inflorescence
Protein (mg/g)	111.06 ± 1.16 ^b^	125.27 ± 1.98 ^a^	101.31 ± 1.11 ^c^	122.13 ± 1.78 ^a^
Carbohydrates (mg/g)	125.42 ± 1.22 ^a^	105.61 ± 1.34 ^c^	98.07 ± 1.45 ^d^	117.54 ± 1.09 ^b^
Lipids (mg/g)	28.10 ± 1.53 ^c^	38.25 ± 0.97 ^a^	21.14 ± 1.08 ^d^	33.22 ± 1.23 ^b^
Total amino acids (mg/g)	0.350 ± 0.005 ^d^	0.632 ± 0.007 ^b^	0.830 ± 0.003 ^a^	0.548 ± 0.006 ^c^
Phenolics (mg/g)	7.34 ± 0.98 ^c^	13.42 ± 1.03 ^b^	29.32 ± 1.42 ^a^	8.32 ± 1.18 ^b^
Nitrogen (%)	1.34 ± 0.19 ^b^	2.38 ± 0.32 ^a^	1.03 ± 0.28 ^c^	2.01 ± 0.15 ^a^
Water content (%)	81 ± 3.15 ^a^	73 ± 2.96 ^b^	39 ± 1.61 ^c^	76 ± 3.06 ^b^
W/N ratio (%)	60.45	30.67	37.86	38.00
Phosphorus (%)	0.29 ± 0.009 ^c^	0.38 ± 0.007 ^a^	0.24 ± 0.003 ^d^	0.32 ± 0.005 ^b^
Potassium (%)	1.25 ± 0.03 ^b^	1.36 ± 0.05 ^ab^	1.28 ± 0.07 ^b^	1.42 ± 0.04 ^a^
Cardiac glycosides (μg/100 mg dry wt.)	123.74 ± 2.32 ^d^	270.56 ± 1.02 ^b^	302.81 ± 2.48 ^a^	208.39 ± 2.14 ^c^
Fibre (%)	5.05 ± 0.61 ^c^	9.81 ± 0.46 ^b^	17.43 ± 0.74 ^a^	3.02 ± 0.97 ^d^

Note: Within a row means followed by different lowercase letters are significantly different at 5% level by DMRT.

**Table 8:** Free fatty acid profile of wild giant milkweed *C. gigantea* (% weight of total fatty acids).

Common Name	Abbreviation	Mature Leaves (wt%)	Inflorescence (wt%)	Senescent (wt%) Leaves	Young Leaves (wt%)
Palmitic acid	C16:0	15.5 ± 1.2 ^a^	11.2 ± 1.1 ^b^	8.7 ± 0.9 ^c^	0.0 ± 0.0 ^d^
Palmitoleic acid	C16:1	0.3 ± 0.02 ^a^	0.01 ± 0.001 ^b^	0.00 ± 0.00 ^b^	0.0 ± 0.0 ^b^
Stearic acid	C18:0	10.5 ± 0.9 ^a^	8.6 ± 0.7 ^b^	4.1 ± 0.4 ^c^	0.0 ± 0.0 ^d^
Oleic acid	C18:1 d9	30.3 ± 2.1 ^a^	26.4 ± 1.8 ^b^	19.2 ± 1.5 ^c^	6.6 ± 0.8 ^d^
Asclepic acid	C18:1 d11	0.8 ± 0.06 ^a^	0.2 ± 0.02 ^b^	0.003 ± 0.000 ^c^	0.0 ± 0.0 ^c^
Linoleic acid	C18:2	36.3 ± 2.5 ^a^	29.6 ± 2.1 ^b^	22.5 ± 1.8 ^c^	4.1 ± 0.5 ^d^
Linolenic acid	C18:3	0.8 ± 0.06 ^a^	0.3 ± 0.03 ^b^	0.1 ± 0.01 ^c^	0.0 ± 0.0 ^d^
Arachidic acid	C20:0	0.6 ± 0.04 ^a^	0.1 ± 0.01 ^b^	0.00 ± 0.00 ^b^	0.0 ± 0.0 ^b^
Behenic acid	C22:0	0.1 ± 0.01 ^a^	0.001 ± 0.000 ^b^	0.00 ± 0.00 ^b^	0.0 ± 0.00 ^b^
Lignoceric acid	C24:0	0.4 ± 0.03 ^a^	0.02 ± 0.02 ^b^	0.001 ± 0.000 ^c^	0.0 ± 0.0 ^d^
Total		95.6 ± 0.8 ^a^	76.43 ± 1.2 ^b^	54.60 ± 1.5 ^c^	10.7 ± 0.5 ^d^

Note: Within a row, means followed by different lowercase letters are significantly different at the 5% level by DMRT. Values represent mean ± S.E.M. (*n* = 3 tissue samples per tissue type).

**Table 9:** Free amino acid profiles (µ moles/g of dry matter) of wild giant milkweed *C. gigantea*.

Amino Acids	Young Leaves	Mature Leaves	Senescent Leaves	Inflorescence
Phosphoserine	0.453	0.496	0.580	0.47
Taurine	0.373	0.309	0.233	0.33
Phosphoethanolamine	0.000	0.188	0.000	0.01
Urea	18.346	32.124	54.115	0.37
Aspartic acid	7.285	4.574	0.480	8.10
Hydroxyproline	0.000	13.769	0.000	7.31
Threonine	0.238	0.568	0.952	0.38
Serine	1.485	1.826	2.380	1.25
Asparagine	77.508	34.870	4.119	51.72
Glutamic acid	11.215	29.982	10.203	27.70
Glutamine	8.611	29.397	9.017	15.02
Amino Adipic Acid	0.457	0.634	0.000	0.53
Proline	22.555	26.884	0.000	24.44
Glycine	1.706	1.370	0.442	1.51
Alanine	10.864	13.188	1.931	12.08
Amino-n-butyric Acid	0.586	1.237	0.000	0.91
Valine	20.001	23.776	6.037	21.71
Cystine	1.183	0.771	0.467	1.10
Methionine	0.035	0.018	0.067	0.031
Cystathionine	0.394	0.238	0.152	0.35
Isoleucine	7.366	7.642	4.271	6.13
Leucine	7.417	6.898	4.050	7.62
Tyrosine	1.926	1.106	0.623	1.55
Phenylalanine	7.141	11.539	3.243	5.07
β-Alanine	3.537	0.985	0.411	2.44
β-Aminoisobutylic Acid	0.027	0.020	0.017	0.011
γ-Aminobutyric Acid	14.105	17.693	3.700	12.21
Ethanolamine	4.237	2.526	1.577	3.55
Ammonia	1.577	3.924	3.415	0.9
Hydroxylysine	0.005	0.003	0.000	0.004
Ornithine	0.017	0.000	0.007	0.00
Lysine	0.523	0.516	0.190	0.48
Histidine	3.338	4.918	1.082	2.89
3-Methylhistidine	0.000	0.008	0.004	0.00
Arginine	2.106	2.165	0.277	2.05

**Table 10:** Phytocomponents present in the GC-MS analysis of young leaves extract from wild giant milkweed *C. gigantea*.

S. No	RT	Compound Name	Formula	Area%–T
1	6.3398	Decane	C_10_H_22_	1.78
2	6.9079	Trichloroacetic acid, 2-ethylhexyl ester	C_10_H_17_Cl_3_O_2_	0.30
3	7.1591	Cyclotrisiloxane, hexamethyl-	C_6_H_18_O_3_Si_3_	0.12
4	8.7250	Cyclopentasiloxane, decamethyl-	C_10_H_30_O_5_Si_5_	0.23
5	9.3331	4-Benzyloxy-3-nitromethyl-pentanoic acid, methyl ester	C_14_H_19_NO_5_	0.48
6	11.2048	Cyclohexasiloxane, dodecamethyl-	C_12_H_36_O_6_Si_6_	0.30
7	13.3242	2,4-Di-tert-butylphenol	C_14_H_22_O	0.39
8	13.4370	Cycloheptasiloxane, tetradecamethyl-	C_14_H_42_O_7_Si_7_	0.45
9	13.6774	2,4-Di-tert-butylphenol	C_14_H_22_O	3.38
10	14.7771	[3-(Trifluoromethyl)-1H-pyrazol-1-yl]acetic acid	C_6_H_5_F_3_N_2_O_2_	0.40
11	15.1267	4-(4-tert-Butylphenyl)-1,3-thiazol-2-ylamine	C_13_H_16_N_2_S	0.34
12	15.4362	2,4-Dihydroxybenzoic acid, 3TMS derivative	C_16_H_30_O_4_Si_3_	0.16
13	15.9569	Hexadecane, 2,6,10,14-tetramethyl-	C_20_H_42_	0.59
14	16.8673	s-Triazine, 2-amino-4-(piperidinomethyl)-4-piperidino-	C_14_H_24_N_6_	0.57
15	17.2606	s-Triazine, 2-amino-4-(piperidinomethyl)-4-piperidino-	C_14_H_24_N_6_	0.67
16	17.3261	Clonitazene	C_20_H_23_ClN_4_O_2_	0.29
17	17.4281	Tetradecane, 2,6,10-trimethyl-	C_17_H_36_	0.40
18	17.5774	Phytol	C_20_H_40_O	0.23
19	17.8614	2-Oxotetrahydrofuryl-5-hexanoic acid, methyl ester	C_11_H_18_O_4_	0.06
20	18.0763	Geranyl isovalerate	C_15_H_26_O_2_	0.16
21	18.1127	Eicosane, 10-methyl-	C_21_H_44_	0.09
22	18.2074	Hexadecanoic acid, methyl ester	C_17_H_34_O_2_	7.03
23	18.2547	7,9-Di-tert-butyl-1-oxaspiro(4,5)deca-6,9-diene-2,8-dione	C_17_H_24_O_3_	11.29
24	18.6189	Ethanol, 2-(tetradecyloxy)-	C_16_H_34_O_2_	0.09
25	18.6662	1,4-Dibutyl benzene-1,4-dicarboxylate	C_16_H_22_O_4_	1.84
26	19.5183	Tetradecane, 2,6,10-trimethyl-	C_17_H_36_	0.21
27	19.8533	9,12-Octadecadienoic acid (Z,Z)-, methyl ester	C_19_H_34_O_2_	4.84
28	19.9225	9,12,15-Octadecatrienoic acid, methyl ester, (Z,Z,Z)-	C_19_H_32_O_2_	23.57
29	20.0340	Phytol	C_20_H_40_O	32.36
30	20.2393	3-(2-Aminoethyl)-2-methyl-1H-indole-5-carbonitrile, 2TMS (isomer 2)	C_18_H_29_N_3_Si_2_	0.65
31	21.1097	Glycine, N-(N-glycyl-L-leucyl)-	C_10_H_19_N_3_O_4_	1.60
32	21.5321	1-Cyclohexyldimethylsilyloxy-3,5-dimethylbenzene	C_16_H_26_OSi	0.99
33	22.1329	1-Cyclohexyldimethylsilyloxy-3,5-dimethylbenzene	C_16_H_26_OSi	3.54
34	22.7702	2-{Bis[4-(dimethylamino)phenyl]methyl}phenol, TMS derivative	C_26_H_34_N_2_OSi	0.59

**Table 11:** Phytocomponents present in the GCMS analysis of mature leaves extract from wild giant milkweed *C. gigantea*.

S. No	RT	Compound Name	Formula	Area%–T
1	5.7316	Vanillin, TBDMS derivative	C_14_H_22_O_3_Si	0.25
2	6.3471	1,1,3,3,5,5,7,7-Octamethyl-7-(2-methylpropoxy)tetrasiloxan-1-ol	C_12_H_34_O_5_Si_4_	1.84
3	6.4199	3,5-Dinitrobenzyl alcohol, TBDMS derivative	C_13_H_20_N_2_O_5_Si	0.87
4	6.9151	Propanoic acid, 2,2-dimethyl-, octyl ester	C_13_H_26_O_2_	0.87
5	7.2101	6,6,8,8,10,10-Hexamethyl-2,5,7,9,11,14-hexaoxa-6,8,10-trisilapentadecane	C_12_H_32_O_6_Si_3_	1.14
6	7.3266	1,2-Benzisothiazol-3(2H)-one, 4-amino-2-methyl-6-nitro-	C_8_H_7_N_3_O_3_S	0.48
7	9.2857	Bis[bicyclo[3.2.0]hept-2-en-4-yl]ether	C_14_H_18_O	0.81
8	11.2012	Cyclohexasiloxane, dodecamethyl-	C_12_H_36_O_6_Si_6_	0.46
9	12.8835	Melezitose	C_18_H_32_O_16_	24.21
10	13.4407	Cycloheptasiloxane, tetradecamethyl-	C_14_H_42_O_7_Si_7_	1.74
11	13.6774	2,4-Di-tert-butylphenol	C_14_H_22_O	5.18
12	14.7625	Diethyl Phthalate	C_12_H_14_O_4_	1.17
13	15.1230	4-(4-tert-Butylphenyl)-1,3-thiazol-2-ylamine	C_13_H_16_N_2_S	0.83
14	15.4435	Cyclooctasiloxane, hexadecamethyl-	C_16_H_48_O_8_Si_8_	0.27
15	15.9533	Eicosane, 10-methyl-	C_21_H_44_	0.99
16	16.2009	1,3-di-iso-propylnaphthalene	C_16_H_20_	0.44
17	16.2810	1,4-di-iso-propylnaphthalene	C_16_H_20_	0.16
18	16.8673	s-Triazine, 2-amino-4-(piperidinomethyl)-4-piperidino-	C_14_H_24_N_6_	0.97
19	17.2606	s-Triazine, 2-amino-4-(piperidinomethyl)-4-piperidino-	C_14_H_24_N_6_	0.77
20	17.4208	Tetradecane, 2,6,10-trimethyl-	C_17_H_36_	0.27
21	17.8214	1,6-Dioxacycloheptadecan-7-one	C_15_H_28_O_3_	1.04
22	18.2074	Hexadecanoic acid, methyl ester	C_17_H_34_O_2_	2.71
23	18.2547	7,9-Di-tert-butyl-1-oxaspiro(4,5)deca-6,9-diene-2,8-dione	C_17_H_24_O_3_	17.98
24	18.6589	1,2-Benzenedicarboxylic acid, butyl 2-methylpropyl ester	C_16_H_22_O_4_	2.64
25	19.9334	9,12,15-Octadecatrienoic acid, methyl ester, (Z,Z,Z)-	C_19_H_32_O_2_	2.69
26	20.0463	Phytol	C_20_H_40_O	16.49
27	20.2393	1-(2-Methylsulfanyl-ethyl)-2,8,9-trioxa-5-aza-1-silabicyclo[3.3.3]undecane	C_9_H_19_NO_3_SSi	1.08
28	21.0951	Glycine, N-(N-glycyl-L-leucyl)-	C_10_H_19_N_3_O_4_	2.75
29	21.5320	1-Cyclohexyldimethylsilyloxy-3,5-dimethylbenzene	C_16_H_26_OSi	1.54
30	22.1365	1-Cyclohexyldimethylsilyloxy-3,5-dimethylbenzene	C_16_H_26_OSi	5.60
31	22.3732	3,3-Bis{4-[(trimethylsilyl)oxy]phenyl}-2-benzofuran-1(3H)-one	C_26_H_30_O_4_Si_2_	0.69
32	22.7702	4-Amino-3-[3-(trifluoromethyl)phenyl]-1,2-thiazole-5-carboxylic acid, 2TMS	C_17_H_23_F_3_N_2_O_2_SSi_2_	1.07

**Table 12:** Phytocomponents present in the GCMS analysis of senescent leaves extract from wild giant milkweed *C. gigantea*.

S. No	RT	Compound Name	Formula	Area%–T
1	6.3435	Decane	C_10_H_22_	2.11
2	6.4090	Methyl 4-methoxysalicylate, TBDMS derivative	C_15_H_24_O_4_Si	1.03
3	6.9079	1-Octyn-3-ol, 4-ethyl-	C_10_H_18_O	0.76
4	9.3186	Bis[bicyclo[3.2.0]hept-2-en-4-yl]ether	C_14_H_18_O	1.31
5	9.4643	dl-2,3-Bis[hexadecyloxy]iodopropane	C_35_H_71_IO_2_	1.05
6	11.2012	Cyclohexasiloxane, dodecamethyl-	C_12_H_36_O_6_Si_6_	0.33
7	13.0001	Melezitose	C_18_H_32_O_16_	4.45
8	13.6738	2,4-Di-tert-butylphenol	C_14_H_22_O	7.66
9	14.7699	Phthalic acid, 3-methylbenzyl tetradecyl ester	C_30_H_42_O_4_	0.66
10	14.9119	4-Hydroxy-4-(3-hydroxybut-1-enyl)-3,5,5-trimethylcyclohex-2-en-1-one, acetate	C_15_H_22_O_4_	0.47
11	15.1268	4-(4-tert-Butylphenyl)-1,3-thiazol-2-ylamine	C_13_H_16_N_2_S	1.51
12	15.4436	Cyclooctasiloxane, hexadecamethyl-	C_16_H_48_O_8_Si_8_	0.25
13	15.7859	1,7-di-iso-propylnaphthalene	C_16_H_2_0	0.48
14	15.9461	Eicosane, 10-methyl-	C_21_H_44_	1.71
15	16.2010	1,3-di-iso-propylnaphthalene	C_16_H_2_0	0.81
16	16.4231	Pentadecane	C_15_H_3_2	0.31
17	16.6817	Glutaric acid, tridec-2-yn-1-yl 2-ethylphenyl ester	C_26_H_38_O_4_	0.79
18	16.8674	s-Triazine, 2-amino-4-(piperidinomethyl)-4-piperidino-	C_14_H_24_N_6_	1.12
19	17.2607	s-Triazine, 2-amino-4-(piperidinomethyl)-4-piperidino-	C_14_H_24_N_6_	1.03
20	17.3226	Undec-10-ynoic acid, tetradecyl ester	C_25_H_46_O_2_	0.74
21	17.4245	Pentadecane, 7-(bromomethyl)-	C_16_H_33_Br	0.57
22	17.5775	Z-10-Methyl-11-tetradecen-1-ol propionate	C_18_H_34_O_2_	0.40
23	17.7632	4,11-Dimethyl-8-(propan-2-yl)-5,12-dioxatricyclo[9.1.0.04,6]dodecan-7-ol, Me	C_16_H_28_O_3_	0.21
24	18.0764	Hexadecane, 2,6,10,14-tetramethyl-	C_20_H_42_	0.21
25	18.2075	Hexadecanoic acid, methyl ester	C_17_H_34_O_2_	2.71
26	18.2548	7,9-Di-tert-butyl-1-oxaspiro(4,5)deca-6,9-diene-2,8-dione	C_17_H_24_O_3_	22.76
27	18.6663	1,4-Dibutyl benzene-1,4-dicarboxylate	C_16_H_22_O_4_	3.29
28	19.5220	Eicosane, 1-iodo-	C_20_H_41_I	0.36
29	19.9444	9,12,15-Octadecatrienoic acid, (Z,Z,Z)-	C_18_H_30_O_2_	2.21
30	20.0391	Phytol	C_20_H_40_O	18.16
31	20.2394	2-(Phenyl-piperidin-1-yl-methyl)-cyclohexanol	C_18_H_27_NO	3.97
32	21.5321	1-Cyclohexyldimethylsilyloxy-3,5-dimethylbenzene	C_16_H_26_OSi	2.61
33	22.0456	Heptadecane, 9-octyl-	C_25_H_52_	1.03
34	22.1366	1-Cyclohexyldimethylsilyloxy-3,5-dimethylbenzene	C_16_H_26_OSi	9.80
35	22.3733	4-Amino-3-[3-(trifluoromethyl)phenyl]-1,2-thiazole-5-carboxylic acid, 2TMS	C_17_H_23_F_3_N_2_O_2_SSi_2_	0.81
36	22.7666	2-{Bis[4-(dimethylamino)phenyl]methyl}phenol, TMS derivative	C_26_H_34_N_2_OSi	1.47
37	22.8285	4-Amino-3-[3-(trifluoromethyl)phenyl]-1,2-thiazole-5-carboxylic acid, 2TMS	C_17_H_23_F_3_N_2_O_2_SSi_2_	0.86

**Table 13:** Phytocomponents present in the GCMS analysis of *C. gigantea* inflorescence.

S. No	RT	Compound Name	Formula	Area%–T
1.	5.3126	Pentanol, 5-amino-	C_5_H_13_NO	0.14
2.	5.4655	4-Pyranone, 2,3-dihydro-	C_5_H_6_O_2_	1.26
3.	5.8916	Acetic acid, 2-phenylethyl ester	C_10_H_12_O_2_	0.21
4.	6.3504	Cyclotetrasiloxane, octamethyl-	C_8_H_24_O_4_Si4	0.39
5.	6.4233	Methyl 4-methoxysalicylate, TBDMS derivative	C_15_H_24_O_4_Si	0.27
6.	6.9258	Valeric acid, dodecyl ester	C_17_H_34_O_2_	0.90
7.	7.1807	Isoquinoline, 4-bromo-	C_9_H_6_BrN	0.67
8.	8.0692	Cyclohexanol, 4-chloro-, trans-	C_6_H_11_ClO	1.81
9.	8.8521	4H-Pyran-4-one, 2,3-dihydro-3,5-dihydroxy-6-methyl-	C_6_H_8_O_4_	0.61
10.	9.2782	L-(-)-Fucose, tetrakis(trifluoroacetate), benzyloxime (isomer 1)	C_21_H_15_F_12_NO_9_	0.74
11.	9.3838	2-(2,6-Dimethylmorpholin-4-yl)ethan-1-ol	C_8_H_17_NO_2_	0.86
12.	9.7261	Cyclotetrasiloxane, octamethyl-	C_8_H_24_O_4_Si4	0.23
13.	9.8499	5-hydroxy-7-methoxyflavanone, tert.-butyldimethylsilyl ether	C_22_H_28_O_4_Si	0.12
14.	10.6037	1,5,9-Cyclododecanetriol	C_12_H_24_O_3_	0.24
15.	11.2591	Phenol, 5-ethenyl-2-methoxy-	C_9_H_10_O_2_	3.58
16.	13.0216	1,3-Propanediol, 2-ethyl-2-(hydroxymethyl)-	C_6_H_14_O_3_	52.08
17.	13.4368	Cycloheptasiloxane, tetradecamethyl-	C_14_H_42_O_7_Si_7_	0.79
18.	13.6698	2,4-Di-tert-butylphenol	C_14_H_22_O	1.86
19.	14.7368	Diethyl Phthalate	C_12_H_14_O_4_	0.34
20.	15.1228	4-(4-tert-Butylphenyl)-1,3-thiazol-2-ylamine	C_13_H_16_N_2_S	0.18
21.	15.4396	2,4-Dihydroxybenzoic acid, 3TMS derivative	C_16_H_30_O_4_Si3	0.14
22.	15.9494	Hexadecane, 2,6,10,14-tetramethyl-	C_20_H_42_	0.30
23.	16.1023	Methyl tetradecanoate	C_15_H_30_O_2_	0.45
24.	16.8634	Eslicarbazepine acetate, N-trimethylsilyl-	C_20_H_24_N_2_O_3_Si	0.45
25.	17.1802	Cyclononasiloxane, octadecamethyl-	C_18_H_54_O_9_Si_9_	0.23
26.	17.2603	s-Triazine, 2-amino-4-(piperidinomethyl)-4-piperidino-	C_14_H_24_N_6_	0.55
27.	18.1962	Hexadecanoic acid, methyl ester	C_17_H_34_O_2_	2.82
28.	18.2508	7,9-Di-tert-butyl-1-oxaspiro(4,5)deca-6,9-diene-2,8-dione	C_17_H_24_O_3_	5.74
29.	18.6441	1,4-Dibutyl benzene-1,4-dicarboxylate	C_16_H_22_O_4_	1.65
30.	18.7351	Octasiloxane, 1,1,3,3,5,5,7,7,9,9,11,11,13,13,15,15-hexadecamethyl-	C_16_H_50_O_7_Si_8_	0.08
31.	19.4234	Ethyl 3-((2R,5S)-7-(cyclohexylsulfonyl)-1-oxa-7-azaspiro[4.5]decan-2-yl)propanoate	C_19_H_33_NO_5_S	0.12
32.	19.8494	9,12-Octadecadienoic acid (Z,Z)-, methyl ester	C_19_H_34_O_2_	2.48
33.	19.8968	9-Octadecenoic acid (Z)-, methyl ester	C_19_H_36_O_2_	4.00
34.	20.0278	Phytol	C_20_H_40_O	0.60
35.	20.1152	Heptadecanoic acid, 16-methyl-, methyl ester	C_19_H_38_O_2_	0.30
36.	20.2354	2,8,9-Trioxa-5-aza-1-silabicyclo[3.3.3]undecane, 1-ethyl-	C_8_H_17_NO_3_Si	1.10
37.	21.0620	Pent-4-enoylamide, 2-methyl-N-(2-butyl)-N-(3-methylbutyl)-	C_15_H_29_NO	1.40
38.	21.5281	1-Cyclohexyldimethylsilyloxy-3,5-dimethylbenzene	C_16_H_26_OSi	0.54
39.	22.1326	1-Cyclohexyldimethylsilyloxy-3,5-dimethylbenzene	C_16_H_26_OSi	1.92
40.	22.8318	4-(4-Amino-3-nitrobenzyl)-2-nitrophenylamine, 2 TMS derivative	C_19_H_28_N_4_O_4_Si2	0.22
41.	25.1842	1,4-Benzenedicarboxylic acid, bis(2-ethylhexyl) ester	C_24_H_38_O_4_	4.25
42.	26.5388	5-Cholesten-3.beta.-ol-7-one, methyl ether	C_28_H_46_O_2_	3.36

## Data Availability

The original contributions presented in this study are included in the article and Supplementary Material. Further inquiries can be directed to the corresponding authors.

## Supplementary Materials

The following supporting information can be downloaded at: https://www.mdpi.com/article/10.3390/insects17090939/s1, Table S1:The sex-specific adult longevity of *Poecilocerus pictus* and *Danaus chrysippus* across tissue treatments of *Calotropis gigantea*.

## Abbreviations

The following abbreviations are used in this manuscript:

AD	Approximate Digestibility
ANOVA	Analysis of Variance
BSA	Bovine Serum Albumin
CI	Consumption Index
DMRT	Duncan’s Multiple Range Test
DNS	Dinitrosalicylic Acid
EC	Enzyme Commission
ECD	Efficiency of Conversion of Digested Food
ECI	Efficiency of Conversion of Ingested Food
FFA	Free Fatty Acid
GC-MS	Gas Chromatography–Mass Spectrometry
GR	Growth Rate
HCl	Hydrochloric Acid
H_2_SO_4_	Sulfuric Acid
NaOH	Sodium Hydroxide
NIST	National Institute of Standards and Technology
RGR	Relative Growth Rate
S.E.M.	Standard Error of the Mean
TCA	Trichloroacetic Acid
TMS	Trimethylsilyl
W/N	Water-to-Nitrogen Ratio
