# Supplementary material for: Host Plant Biochemical Influence on the Foraging and Food Utilization of Poecilocerus pictus and Danaus chrysippus on Calotropis gigantea: A Behavioral Ecology Approach
Source: Insects. 2026 Sep 8;17(9):939. doi: 10.3390/insects17090939 (PMC13607906; doi:10.3390/insects17090939)
Supplement: Supplementary file 1 [file insects-17-00939-s001.zip › insects-4498723-supplementary.pdf]

Table S1 The sex-specific adult longevity of *Poecilocerus pictus* and *Danaus chrysippus* across tissue treatments of *Calotropis gigantea*

Species	Tissue Type	Male Longevity (days)	Female Longevity (days)	ANOVA (Sex × Tissue)
P. pictus	Inflorescence	95.35 ± 1.23	118.50 ± 1.34	$F_{3,72}$ = 1.24, P = 0.302
	Young Leaves	104.55 ± 1.87	127.39 ± 1.92	$F_{3,72}$ = 1.18, P = 0.324
	Mature Leaves	116.23 ± 1.11	140.70 ± 1.00	$F_{3,72}$ = 1.45, P = 0.235
	Senescent Leaves	88.76 ± 1.16	109.84 ± 1.18	$F_{3,72}$ = 1.33, P = 0.271
D. chrysippus	Inflorescence	8.34 ± 1.67	8.72 ± 1.23	$F_{3,72}$ = 0.98, P = 0.407
	Young Leaves	9.56 ± 1.23	10.48 ± 1.69	$F_{3,72}$ = 1.05, P = 0.376
	Mature Leaves	5.34 ± 1.14	4.78 ± 1.23	$F_{3,72}$ = 0.89, P = 0.451
	Senescent Leaves	3.38 ± 1.14	3.34 ± 1.37	$F_{3,72}$ = 0.76, P = 0.521

Note: Values represent mean ± S.E.M. (n = 10 pairs per treatment per species). Non-significant interaction terms (P > 0.05 for all comparisons) confirm that treatment effects on longevity are consistent across sexes, and sex did not confound the observed dietary effects."
